# Bacterial cell wall nanoimaging by autoblinking microscopy

**DOI:** 10.1038/s41598-018-32335-z

**Published:** 2018-09-19

**Authors:** Kevin Floc’h, Françoise Lacroix, Liliana Barbieri, Pascale Servant, Remi Galland, Corey Butler, Jean-Baptiste Sibarita, Dominique Bourgeois, Joanna Timmins

**Affiliations:** 1 Univ. Grenoble Alpes, CEA, CNRS, IBS, F-38000 Grenoble, France; 2 Institute for Integrative Biology of the Cell (I2BC), CEA, CNRS, Univ. Paris-Sud, Université Paris-Saclay, 91198 Gif-sur-Yvette, France; 3 Institut Interdisciplinaire de Neurosciences, University of Bordeaux, Bordeaux, France; 4 Centre National de la Recherche Scientifique, UMR5297, Bordeaux, France

## Abstract

Spurious blinking fluorescent spots are often seen in bacteria during single-molecule localization microscopy experiments. Although this ‘autoblinking’ phenomenon is widespread, its origin remains unclear. In *Deinococcus* strains, we observed particularly strong autoblinking at the periphery of the bacteria, facilitating its comprehensive characterization. A systematic evaluation of the contributions of different components of the sample environment to autoblinking levels and the in-depth analysis of the photophysical properties of autoblinking molecules indicate that the phenomenon results from transient binding of fluorophores originating mostly from the growth medium to the bacterial cell wall, which produces single-molecule fluorescence through a Point Accumulation for Imaging in Nanoscale Topography (PAINT) mechanism. Our data suggest that the autoblinking molecules preferentially bind to the plasma membrane of bacterial cells. Autoblinking microscopy was used to acquire nanoscale images of live, unlabeled *D. radiodurans* and could be combined with PALM imaging of PAmCherry-labeled bacteria in two-color experiments. Autoblinking-based super-resolved images provided insight into the formation of septa in dividing bacteria and revealed heterogeneities in the distribution and dynamics of autoblinking molecules within the cell wall.

## Introduction

The advent of super-resolution fluorescence imaging has opened considerable opportunities for the investigation of bacteria, notably because the small size of these microorganisms largely prevents their detailed visualization by conventional optical microscopy^1,2^. Practically all nanoscopy schemes, including point-scanning, structured-illumination and single-molecule localization methods have thus been used to provide fundamental insight into complex mechanisms in bacteria such as DNA repair^3,4^, cell division^5^, gene expression^6^ or cell wall synthesis^7^. Localization methods such as PhotoActivated Localization Microscopy (PALM) and direct Stochastic Optical Reconstruction Microscopy (dSTORM) offer the advantages that they typically achieve the highest spatial resolution^8–10^, are able to generate 3-D multicolor images with relatively simple instrumentation^11^, and can deliver both a quantitative^12^ and a dynamic^13^ view of processes under study.

Yet, a potential caveat when these techniques are used for bacterial imaging has recently been reported: several localization microscopy studies of unlabeled bacteria have indeed reported punctate fluorescent spots that were found to be indistinguishable from those originating from single PAmCherry molecules^3,14,15^. These studies revealed that some bacteria, such as *Bacillus subtilis* exhibited higher levels of such fluorescent spots than others such as *Escherichia coli* or *Enterococcus faecalis*. It was proposed that the spurious localization events in *B. subtilis* were associated with membrane localized fluorophores, but only limited details were given concerning the properties of these fluorophores as well as their possible origin^3^.

In the present study, we show that this phenomenon, which we have named ‘autoblinking’, is widespread in bacteria and is observed to varying extents in both Gram-negative and Gram-positive species. Interestingly, two radiation-resistant *Deinococcus* strains, *D. radiodurans* and *D. deserti*, were found to exhibit particularly strong autoblinking located at the periphery of *Deinococcus* cells, as in *B. subtilis*, but at a much higher level. The density of autoblinking was such that it provided nanoscale imaging of the *D. radiodurans* cell wall ‘for free’ in both live and fixed cells. Intrigued by these observations, we investigated the possible origin of the autoblinking molecules, characterized their photophysical properties and demonstrated their potential relevance in deciphering *D. radiodurans* cell wall structure and dynamics.

## Results

### Autoblinking: a widespread phenomenon in bacteria

**Figure 1:**
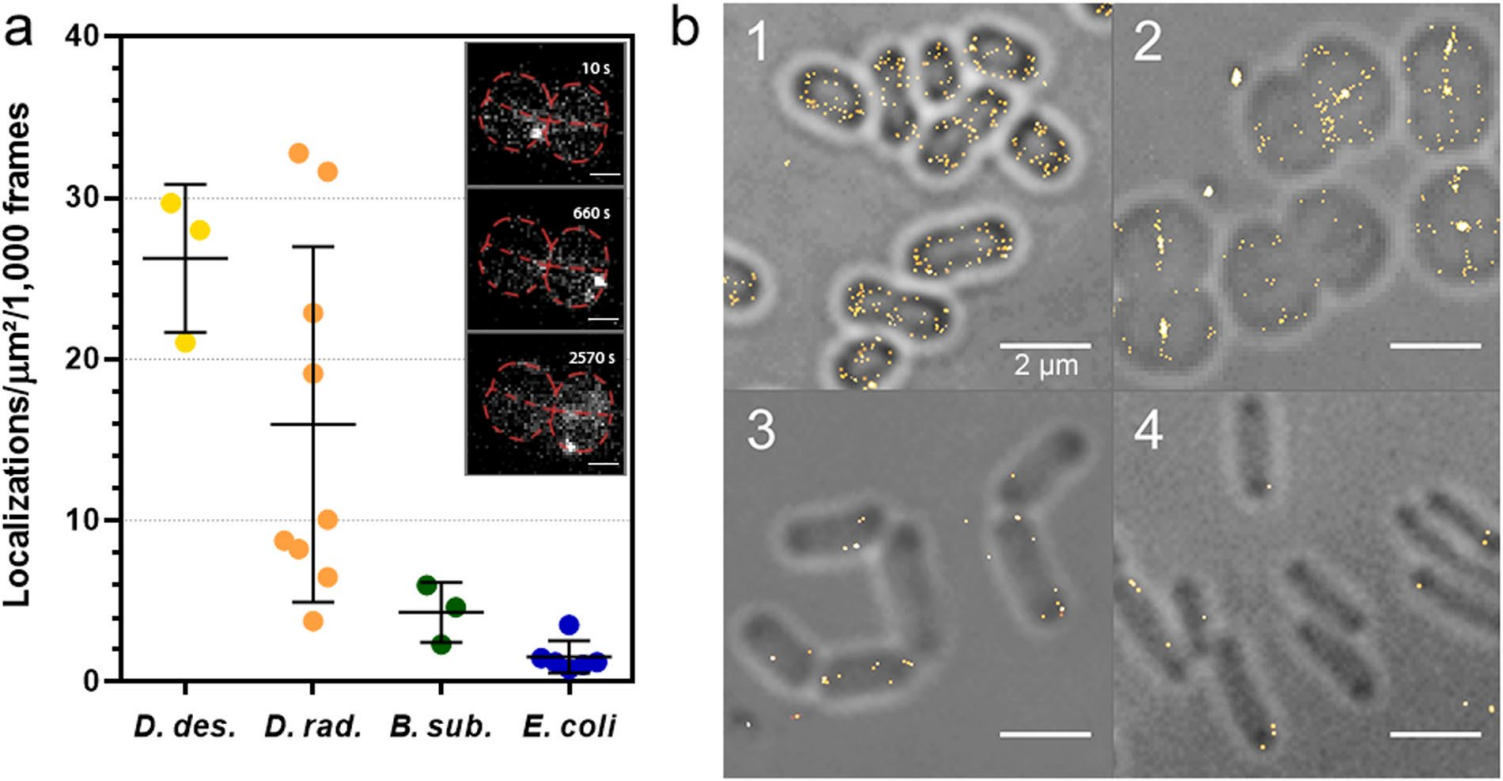
Autoblinking levels in *D. deserti*, *D. radiodurans*, *B. subtilis* and *E. coli*. (**a**) Number of localizations per μm^2^ per 1000 frames extracted from images acquired with 50 ms exposure under continuous 0.8 kW/cm^2^ 561 nm laser. Individual data points correspond to the autoblinking levels derived from a given stack of images. Means and standard deviations are plotted in the graph. Inset: examples of raw autoblinking signal (bright white spots) observed in the cell periphery of a *D. radiodurans* tetrad (outlined in red and presented in Fig. 2) at different timepoints during image acquisition (see also Supplementary Movie S1). Scale bar: 1 μm. (**b**) Representative reconstructions of live, unlabeled *D. deserti* (1), *D. radiodurans* (2), *B. subtilis*. (3) and *E. coli* (4) superimposed on their respective brightfield images. In each case, the reconstructed images are derived from a stack of 1000 frames of 50 ms exposure acquired under continuous 0.8 kW/cm^2^ 561 nm laser. Scale bar: 2 μm.

In order to test whether *D. radiodurans* bacterial cells would be suitable for single-molecule localization microscopy (SMLM) despite their high carotenoid content and associated pink color, we submitted unlabeled bacteria to PALM imaging. Illumination with a 561 nm laser (0.8 kW/cm^2^), in the absence of additional 405 nm light, resulted in rapid fading of the autofluorescence of the bacterial cell wall and progressive appearance of sparse single-molecule blinking events (Fig. 1a and Supplementary Movie S1), which were reminiscent of those described in *B. subtilis* and in *E. coli*^14,15^. We studied this ‘autoblinking’ phenomenon in several bacterial species (*B. subtilis*, *E. coli*, *D. radiodurans* and *D. deserti*) grown in their respective media, and observed that, although always present, its level was clearly species-dependent (Fig. 1). We noticed that the levels of autoblinking were much higher in *Deinococcus* strains than in the model bacteria *B. subtilis* and *E. coli*. While *D. deserti* exhibited the highest levels of autoblinking, *E. coli* showed the lowest level, although both of these bacteria are rod-shaped Gram-negative bacteria. This suggests that the extent of autoblinking is unrelated to the shape and Gram staining of bacteria. Likewise, *D. deserti* and *D. radiodurans* both displayed high levels of autoblinking, although they differ greatly in terms of cell morphology. To further characterize the autoblinking phenomenon, we focused our work on the well-studied *D. radiodurans* bacterium.

### Autoblinking in *D. radiodurans*

**Figure 2:**
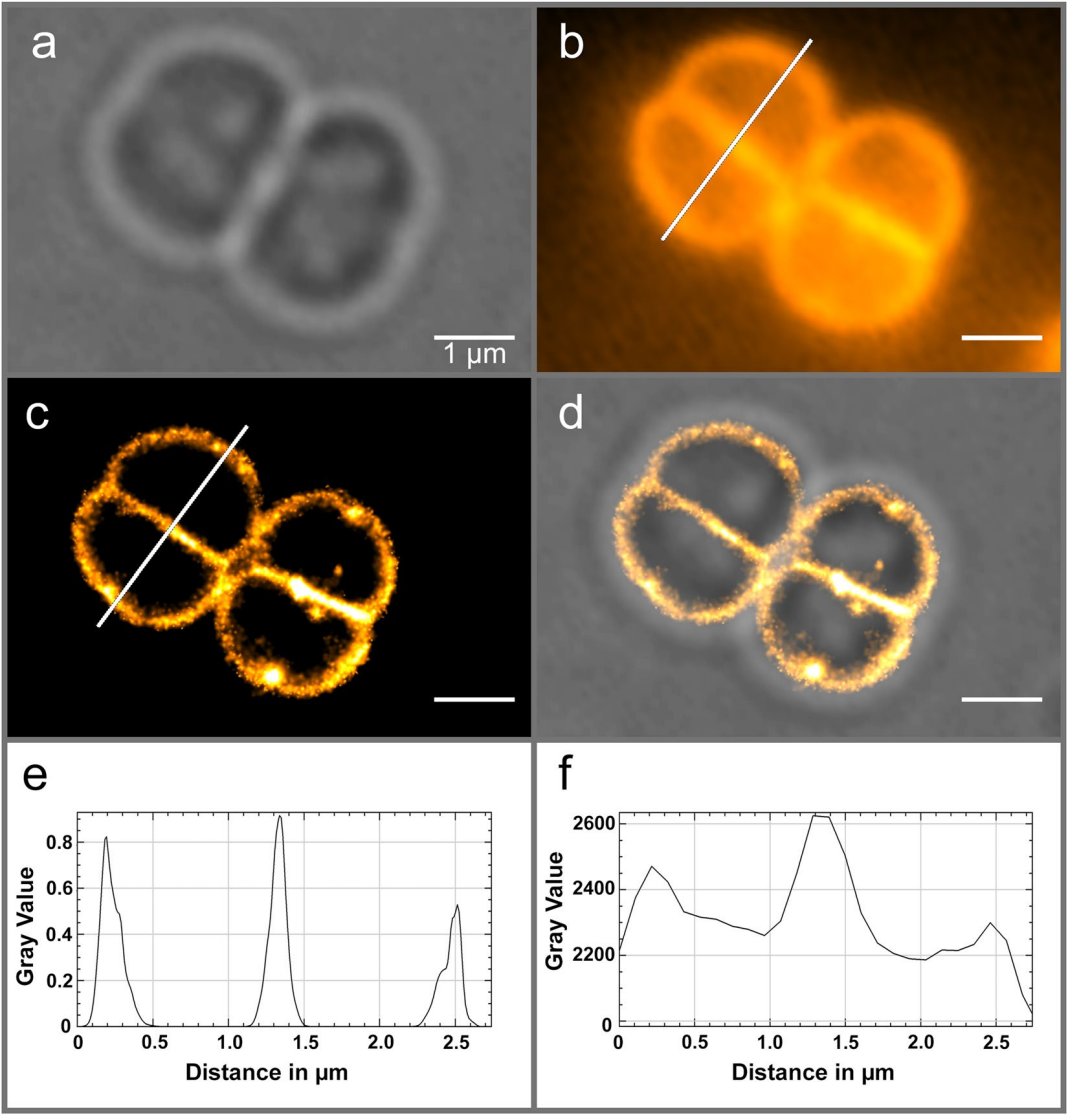
Autoblinking nanoscopy of live, unlabeled *D. radiodurans* bacterial cell walls. (**a**) Brightfield image. (**b**) Z-projection of 1000 frames of the original stack. (**c**) Super-resolved image rendered from a stack of 50,000 frames acquired under continuous 0.8 kW/cm^2^ 561 nm laser (Supplementary Movie S1 provides an example of 500 frames of this stack). (**d**) Superimposed image of (**a**,**c**). (**e**,**f**) Profiles along the white line of (**c**,**b**) respectively. Scale bar: 1 μm.

*D. radiodurans* is a pink-colored, Gram-positive, spherical bacterium able to withstand the normally lethal effects of DNA-damaging agents, notably ionizing radiation, UV light and desiccation^16–18^. As such, specific properties of this microorganism related to this outstanding phenotype, including its morphology, DNA repair repertoire, nucleoid organization, carotenoid content and cell wall structure have been the subject of intense research over the past decades^19–25^. SMLM of live, unlabeled *D. radiodurans* bacteria provided unambiguous, super-resolved images of the bacterial cell wall (Fig. 2), similar to those obtained using the membrane dye Nile Red (Supplementary Fig. S1). Practically no autoblinking (<0.2 localizations per μm^2^/1000 frames) was observed within the cytoplasm or in the extracellular medium. Autoblinking molecules were localized with a mean precision of ~22 nm and the Fourier Ring Correlation (FRC) method^26,27^ suggested an overall resolution of 110 nm. Simulations indicated that the achieved resolution, however, was significantly affected in the 2-D images by 3-D projection of the curved cell wall through the objective’s depth of field, notably on the cytoplasmic side of the external cell border (Fig. 2e, Supplementary Discussion and Fig. S2).

### Source of autoblinking

The finding that autoblinking is particularly strong and localized to the cell wall in *Deinococcus* strains led us to hypothesize that this phenomenon could be associated with the high content in carotenoid molecules of these bacteria, which are responsible for their pink/orange color and in the case of *D. radiodurans* are known to associate with its unusual cell wall^28^. Although *D. radiodurans* is classified as a Gram positive bacterium, it possesses a complex, multilayered cell wall including an inner plasma membrane, a peptidoglycan layer, an interstitial layer, a highly structured ‘S-layer’, and finally a thick layer of carbohydrates^21,29^ (Supplementary Fig. S3). Earlier studies have reported that *D. radiodurans* produces a specific carotenoid, deinoxanthin, and the biosynthesis pathway for this carotenoid has been well characterized^30^ (Supplementary Fig. S4).

**Figure 3:**
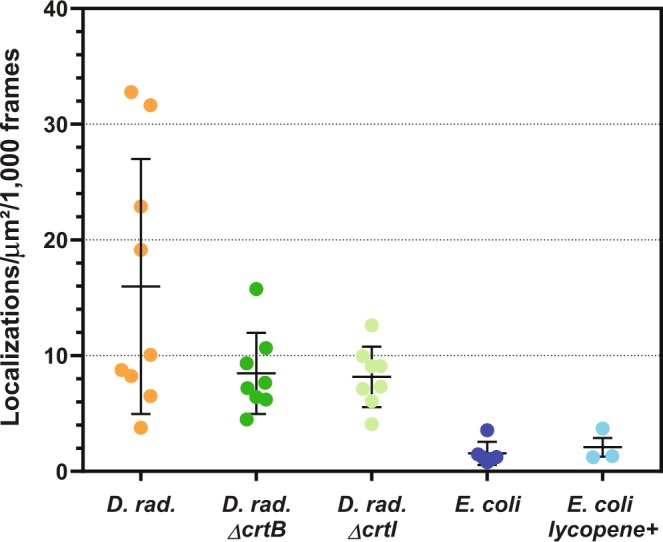
Autoblinking levels (localizations/μm^2^/1000 frames) in wild-type and carotenoid biosynthesis mutants (Δ*crtB* and Δ*crtI*) of *D. radiodurans*, and in wild-type and lycopene producing *E. coli* strains. Images were acquired with a 50 ms frametime under continuous 0.8 kW/cm^2^ 561 nm laser. Individual data points correspond to the autoblinking levels derived from a given stack of images. Means and standard deviations are plotted in the graph.

To test our hypothesis, we evaluated the levels of autoblinking in two mutant strains of *D. radiodurans* (Δ*crtB* and Δ*crtI*), which are colorless and no longer produce complex carotenoids^23^. Δ*crtB* mutant accumulates a carotenoid precursor, geranylgeranyl-pyrophosphate (GGPP), while Δ*crtI* accumulates phytoene. Moreover, we also investigated the autoblinking levels in an engineered *E. coli* K12 strain, BW-LYCO, able to produce high levels of a common carotenoid, lycopene, the pigment that gives the red color to tomatoes^31^. No significant difference was observed between the levels of autoblinking in wild-type (WT), Δ*crtI* and Δ*crtB D. radiodurans*, on the one hand, and between WT and lycopene-producing *E. coli*, on the other hand (Fig. 3). These results strongly suggest that carotenoids are not implicated in the autoblinking phenomenon, also in agreement with the notion that carotenoids in their ground state typically do not absorb 561 nm light strongly^32^.

To determine whether the source of autoblinking is endogenous or exogenous, we evaluated autoblinking levels in exponential or stationary growing live bacteria and in fixed bacteria (Supplementary Fig. S5). Although the cell wall labelling was partly deteriorated in fixed cells, we did not observe any significant difference in the autoblinking levels in live vs. fixed cells. Moreover, autoblinking was found to be very long-lasting (see photophysics analysis below) even in fixed cells, suggesting that the source is likely not endogenous. We, thus, set out to investigate whether exogenous fluorophores could be responsible for autoblinking.

**Figure 4:**
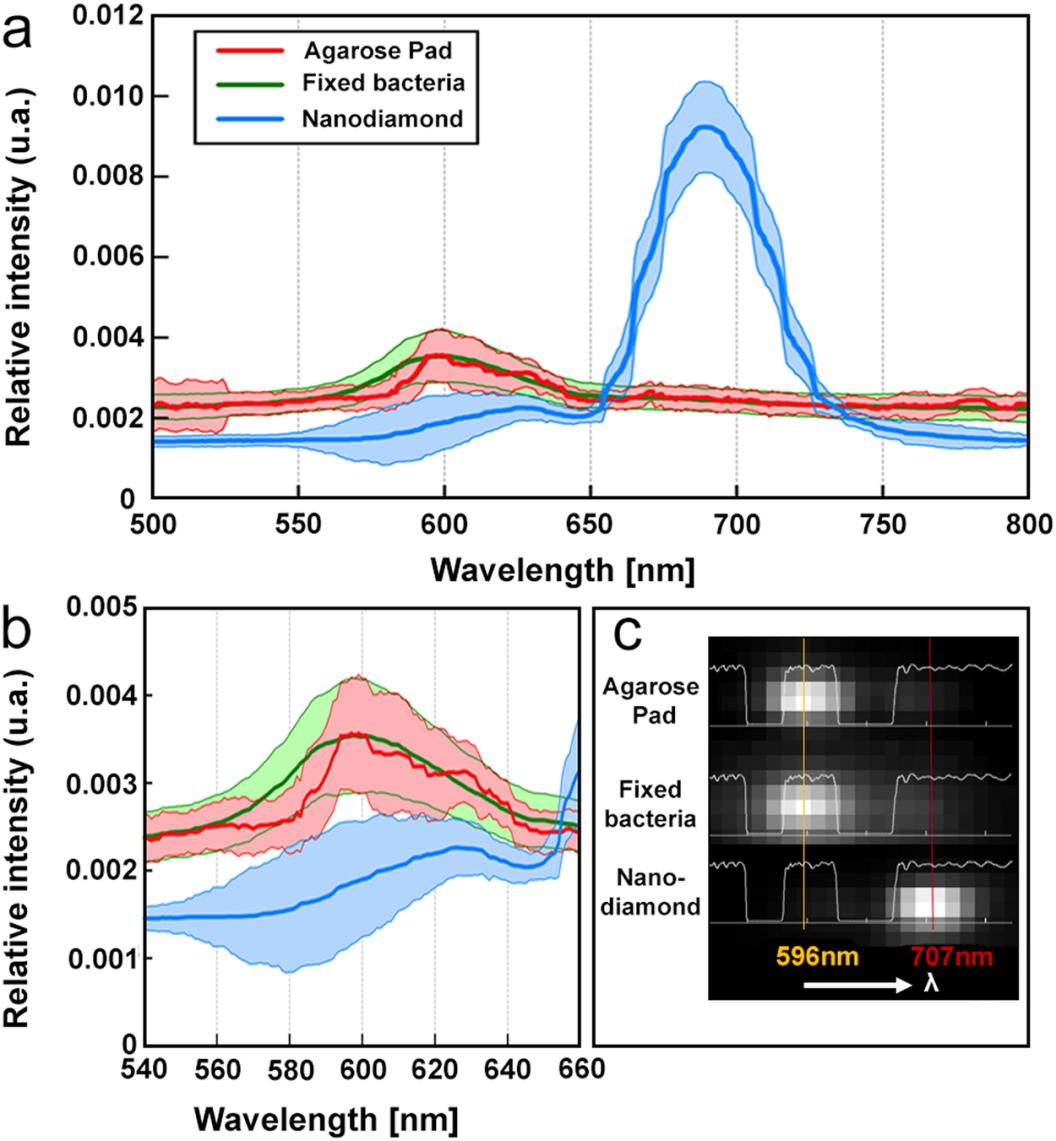
Single-molecule spectral imaging of molecules on the surface of the agarose pads (red), of autoblinking molecules in fixed *D. radiodurans* cells (green), and of nanodiamonds (blue). (**a**) Average emission spectra (500 nm to 825 nm) derived from 34, 1221 and 1013 individual spectra respectively. (**b**) Inset of (**a**) focusing on the 540 nm to 660 nm wavelength range. (**c**) Examples of raw spectra produced by molecules on the agarose pad, within fixed bacterial samples and by nanodiamonds. In light white is represented the Quad band notch spectrum used for the acquisition on the spectral channel.

Under PALM illumination conditions, sparse blinking events can always be seen at the surface of glass coverslips or agarose pads, despite thorough cleaning of the glassware and even in the absence of any deposited biological sample. To investigate whether the molecules at the origin of these blinking events could be the same as those decorating the periphery of *D. radiodurans* cells, we performed a comparative study using single-molecule spectral imaging^33^. We observed that, under illumination with 561-nm light, the average spectral signature of localization events at the *D. radiodurans* cell wall resulting from autoblinking closely matched that derived from localization events detected at the surface of the agarose pad (Fig. 4). This was in sharp contrast with the spectral signature produced by nanodiamonds that were used for drift correction and spectral calibration, which showed a strongly red-shifted spectrum (Fig. 4). These results thus strongly suggest that blinking molecules originating from the sample environment could be the same as those responsible for autoblinking in *D. radiodurans*. Spectral imaging was also performed on sparse molecules seen directly at the surface of untreated glass coverslides (Supplementary Fig. S6). The spectrum of such molecules displayed a peak centered around 600 nm, similar to the spectra of autoblinking molecules, but also exhibited a second red-shifted peak (around 675 nm), indicating that the nature of these molecules may differ from those found in the cell walls of the bacteria and on the agarose pad surface.

**Figure 5:**
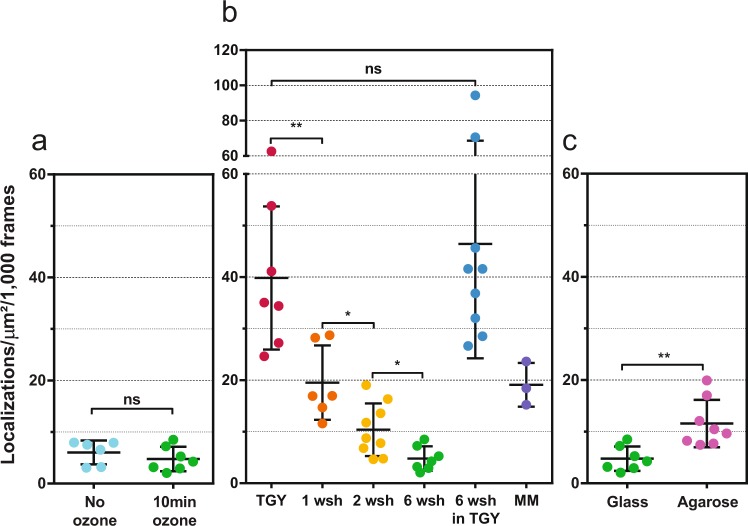
Comparative study of the autoblinking levels in fixed, unlabeled *D. radiodurans* cells. (**a**) Effect of ozone treatment of glass coverslides on the number of localizations in cells per µm^2^ per 1000 frames. (**b**) Effect of TGY growth medium and washes with highly pure PBS prior to sample mounting on the number of localizations in cells per µm^2^ per 1000 frames. TGY grown cells were either deposited directly between coverslides (TGY) or were washed once (1 wsh), twice (2 wsh) or 6 times (6 wsh) prior to imaging. A sample of cells washed 6 times with PBS was also resuspended again in TGY medium (6 wsh in TGY) before imaging. Cells initially grown in TGY and then transferred to minimal medium (MM) for 24 hours were also imaged. (**c**) Effect of the agarose pad on the number of localizations in cells per µm^2^ per 1000 frames. All images were acquired with a 50 ms frametime under continuous 0.8 kW/cm^2^ 561 nm laser. Individual data points correspond to the autoblinking levels derived from a given stack of images. Means and standard deviations are plotted in the graph.

We then systematically investigated possible effects of the different components used for sample preparation on autoblinking levels in *D. radiodurans* (Fig. 5 and Supplementary Figs S6 and S7). In all these experiments, we noticed that the autoblinking levels displayed a relatively high variation from sample to sample and even from one field of view to another within a given sample.

We first studied the influence of the glass coverslides on which the samples are deposited. In the absence of preliminary treatment in an ozone oven, we observed numerous blinking events at the surface of the coverslides that rapidly bleached under excitation by the laser beam. These blinking events could be efficiently removed by ozone treatment (Supplementary Fig. S6). Although blinking on glass was generally accompanied by the sparse detection of rapidly diffusing fluorescent molecules in the medium around the imaged bacteria, it did not lead to a significant increase in autoblinking levels within the cells (Fig. 5a). This finding is in line with the observation by spectral imaging that sparse blinking molecules on the glass surface may differ from those binding to *D. radiodurans* cell walls. Nevertheless, as it is generally recommended for SMLM experiments, all our experiments made use of ozone-treated glass coverslides. Next, we studied the influence of the growth medium on autoblinking levels. These experiments clearly revealed that growing and/or resuspending cells in rich TGY medium resulted in the highest levels of autoblinking, whereas washing TGY-grown cells with highly pure PBS solution significantly reduced autoblinking to a minimal level (Fig. 5b). The main source of autoblinking is thus likely to be the cell culture medium. Growing cells in minimal medium (MM) resulted in relatively low levels of autoblinking, similar to those obtained with washed cells. A comparison of the levels of autoblinking in TGY and MM measured at the surface of agarose pads in the absence of bacterial cells confirmed that TGY is indeed the major source of exogenous fluorophores (Supplementary Fig. S7). Finally, we compared autoblinking levels in cells mounted either directly between glass coverslides or deposited on agarose pads (Fig. 5c). The agarose pad led to slightly increased autoblinking levels, suggesting that the agarose itself may also be a source of autoblinking fluorophores. It is noteworthy that throughout our study, we noticed that conditioning (in glass vs. plastic) and/or age of chemicals used for sample preparation also influenced the autoblinking levels (Supplementary Fig. S7).

Having determined that the growth medium is the principal source of autoblinking molecules, we set out to determine which part of the bacterial cell wall preferentially traps these fluorophores. As illustrated in Supplementary Fig. S3, *D. radiodurans* possesses a complex cell wall composed of multiple layers. The S-layer and carbohydrate layer are only present in the external cell periphery and not in the internal cell septa^21,34^. As we can see in Fig. 2 and Supplementary Fig. S1, autoblinking molecules efficiently label both the external and internal cell walls, indicating that it must be associated with either the peptidoglycan layer and/or the plasma membrane. We thus examined the levels and distribution of autoblinking in *D. radiodurans* cells treated with either lysozyme, an enzyme known to digest both the carbohydrate and the peptidoglycan layers of bacterial cell walls, or with Triton X-100, a common detergent used to disrupt membrane bilayers (Supplementary Fig. S8). The autoblinking distribution was essentially unaffected by lysozyme treatment, while in contrast, Triton X-100 led to a complete loss of cell wall labelling. These findings strongly suggest that autoblinking molecules bind to the plasma membrane, in line with the very similar images obtained when using the lipid binding dye, Nile Red (Supplementary Fig. S1). Although all bacteria possess such a lipidic membrane, the species-dependent levels of autoblinking observed in different bacteria may nonetheless be explained by the particular structure and chemical nature of their cell walls, which may differentially affect accessibility to the plasma membrane.

### Autoblinking mechanism and photophysics

We next set out to characterize the properties of the autoblinking molecules and compare them with those of PAmCherry, a classical photoactivatable red fluorescent protein (PAFP) routinely used in PALM experiments, which we expressed in *D. radiodurans*. Photophysical parameters extracted from the analysis of single-molecule fluorescence traces^35^ are listed in Supplementary Tables S1 and S2 and histograms of the distribution of single-molecule photon counts are provided in Supplementary Fig. S9. Under 561 nm illumination (0.8 kW/cm^2^), autoblinking fluorophores delivered a mean number of photons per localization event approx. equal to that of PAmCherry, while the total photon count was about half that of PAmCherry (Supplementary Table S1). Interestingly, autoblinking fluorophores could be successfully observed upon excitation with 488 nm, 561 nm, and to a lesser extent 643 nm lasers and thus exhibit a wide excitation spectrum (Supplementary Table S2 and Fig. S9).

**Figure 6:**
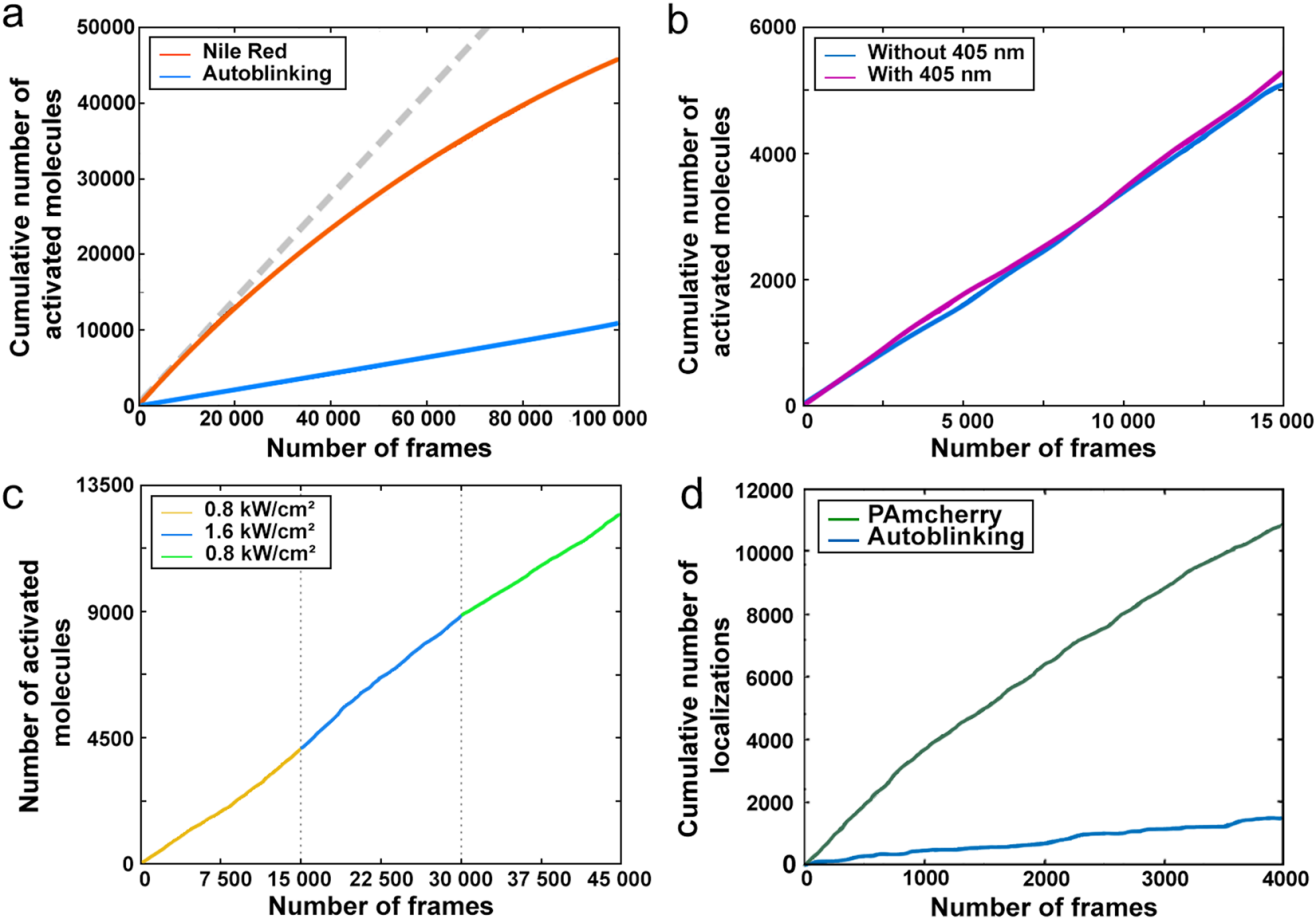
Photophysical properties of autoblinking molecules. (**a**) Cumulative number of autoblinking molecules (blue) under constant 561 nm laser illumination (0.8 kW/cm^2^), compared to Nile Red (orange). Accumulation of autoblinking molecules follows a linear regression, while that of Nile Red progressively slows down during the data acquisition. (**b**) Cumulative number of activated autoblinking molecules under constant 561 nm laser illumination (0.8 kW/cm^2^) in the presence (violet) and absence (blue) of constant 405 nm illumination. (**c**) Cumulative number of activated autoblinking molecules under varying 561 nm laser illumination (0.8 kW/cm^2^, yellow; 1.6 kW/cm^2^, blue; 0.8 kW/cm^2^, green). Accumulation of autoblinking molecules follows a linear progression and is only marginally affected by the increased laser power. (a-c) Images were acquired with a 50 ms timeframe. (**d**) Cumulative number of localizations of cytoplasmic PAmCherry (green) expressed in *D. radiodurans* and of autoblinking molecules (blue). PAmCherry localizations were acquired first using a 5 ms timeframe, constant 561 nm illumination (0.8 kW/cm^2^) and varying 405 nm laser, and once all the PAmCherry molecules had been bleached, the autoblinking molecules were imaged under constant 561 nm illumination (0.8 kW/cm) and with 50 ms timeframes.

In contrast to PAmCherry, the apparent rate of activation of autoblinking molecules was found to be (i) constant overtime, (ii) insensitive to the use of additional 405 nm light, and (iii) independent of the readout laser power (Fig. 6). These observations demonstrate that autoblinking molecules decorate *D. radiodurans* cell walls by transient binding, thus allowing super-resolution imaging through Point Accumulation for Imaging in Nanoscale Topography (PAINT)^36^. This finding was corroborated by the observation that the apparent bleaching rate of autoblinking molecules (~18 s^−1^) was essentially independent of the applied readout laser power (Supplementary Table S3), thus being driven by fluorophore unbinding rather than bleaching or reversible dark state formation.

As the membrane dye Nile Red is also classically used for PAINT imaging, we compared its photophysical properties to those of autoblinking molecules (Supplementary Table S3). To this aim, the concentration of Nile Red was adjusted so that 90% of localizations originated from this dye, and only 10% from autoblinking molecules. Two interesting differences between the two dyes were noticed. Firstly, the average unbinding rate of Nile Red molecules was 15% higher than that of autoblinking molecules. Secondly, contrary to autoblinking molecules, the apparent activation rate of Nile Red molecules progressively decayed along data acquisition (Fig. 6a), suggesting a depletion of the pool of available molecules for binding, possibly associated to a higher susceptibility to photobleaching. Thus the properties of autoblinking molecules make them more favorable for single particle tracking (spt) than Nile Red (see Supplementary Methods).

### PALM imaging in the presence of autoblinking

**Figure 7:**
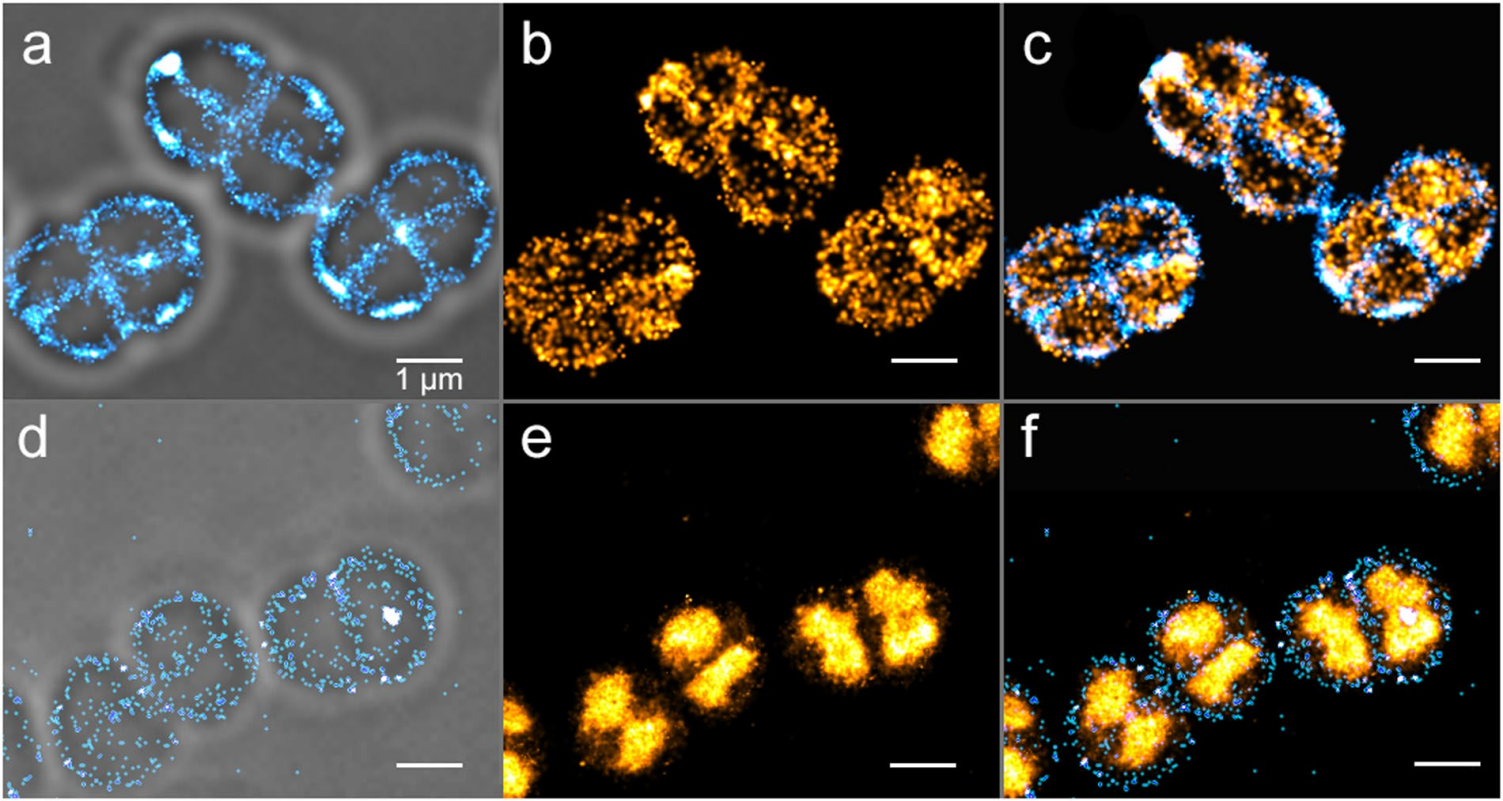
Two-color PALM/PAINT imaging of *D. radiodurans*. (**a**–**c**) PALM/PAINT imaging of *D. radiodurans* cells expressing cytoplasmic PAmCherry. (**a**) Autoblinking-based PAINT image (50 ms frametime; 561 nm laser only), superimposed on the brightfield image. (**b**) PAmCherry-based PALM image (4.8 ms frametime; 561 nm plus 405 nm lasers). **(c**) Superimposed image of (**a**) and (**b**). (**d**–**f**) PALM/PAINT imaging of *D. radiodurans* cells expressing PAmCherry fused to HU. (**d**) Autoblinking-based PAINT image (50 ms frametime; 643 nm laser), superimposed on the brightfield image. (**e**) PAmCherry-based PALM image (50 ms frametime; 561 nm laser). (**f**) Superimposed image of (**d**) and (**e**). Scale bar: 1 μm.

To test whether PALM imaging of a specific molecular target labeled with a photo-transformable fluorescent protein (PTFP) can nonetheless be achieved in the presence of autoblinking, we attempted to overrun the phenomenon by taking advantage of the relatively low binding rate of autoblinking molecules and of their lack of response to a 405 nm laser. We imaged wild-type *D. radiodurans* cells either transformed with a plasmid expressing low levels of PAmCherry or genetically-modified to express the highly abundant nucleoid-associated HU protein^37^ fused to PAmCherry. In the first case, illumination at both 405 nm (0.4 W/cm^2^) and 561 nm (0.8 kW/cm^2^) combined with the use of short frametimes (~5 ms, also serving to freeze the movement of the rapidly diffusing PAmCherry molecules) allowed acquiring high-quality PAmCherry images in less than a minute, at the expense of a reduced field of view but keeping the amount of autoblinking signal sufficiently low (Supplementary Fig. S10). In the second case, “readout activation” by the 561 nm laser alone^38^ was sufficient to rapidly collect a PAmCherry image and outrun autoblinking (Fig. 7).

Next, we set out to investigate whether two-color PALM/PAINT images could be acquired by using both the signals of PAmCherry and that of autoblinking, as reported previously using PAmCherry-labelled proteins and the membrane dye Nile Red in fixed *E. coli* cells^39^. In the case of low PAmCherry expression (Fig. 7a–c), an autoblinking-based image of the bacterial cell wall could be collected before the PAmCherry image by first exciting the sample at 561 nm (0.5 kW/cm^2^), in the absence of 405 nm illumination, and with a standard 50 ms frametime. The PAmCherry image was then collected as described above, using 405 nm illumination and a reduced frametime (~5 ms) compensated by a stronger 561 nm laser power density (2 kW/cm^2^), so as to outrun the binding rate of autoblinking molecules. This data collection strategy shows similarities to that recently proposed for two-color experiments based on primed photoconversion^40^. The resulting two-color image (Fig. 7c), free of chromatic aberrations, suggests that PAmCherry molecules are not uniformly distributed throughout the cytoplasm, but rather tend to accumulate close to the periphery of the cell, possibly as a result of exclusion by the highly condensed nucleoid of *D. radiodurans* cells^20^.

In the case of the highly abundant HU-PAmCherry fusion protein (Fig. 7d–f), strong readout activation by the 561-nm light prevented imaging of autoblinking molecules without significant cross talk with PAmCherry signals. However, we relied on the spectral differences between the autoblinking molecules and PAmCherry to image autoblinking using a 643 nm laser (Fig. 7d). Although not ideal, since autoblinking molecules are rather weakly excited at this wavelength, this strategy provided a two-color image in which the *D. radiodurans* nucleoids could clearly be positioned relative to the surrounding cell wall (Fig. 7f).

Overall, our experiments demonstrate that PALM imaging of a specific target labeled with a PTFP is possible in *D. radiodurans* despite the autoblinking phenomenon, and that under suitable conditions, autoblinking could in fact be used for complementary cell wall imaging in single- or two-color PALM/PAINT experiments.

**Figure 8:**
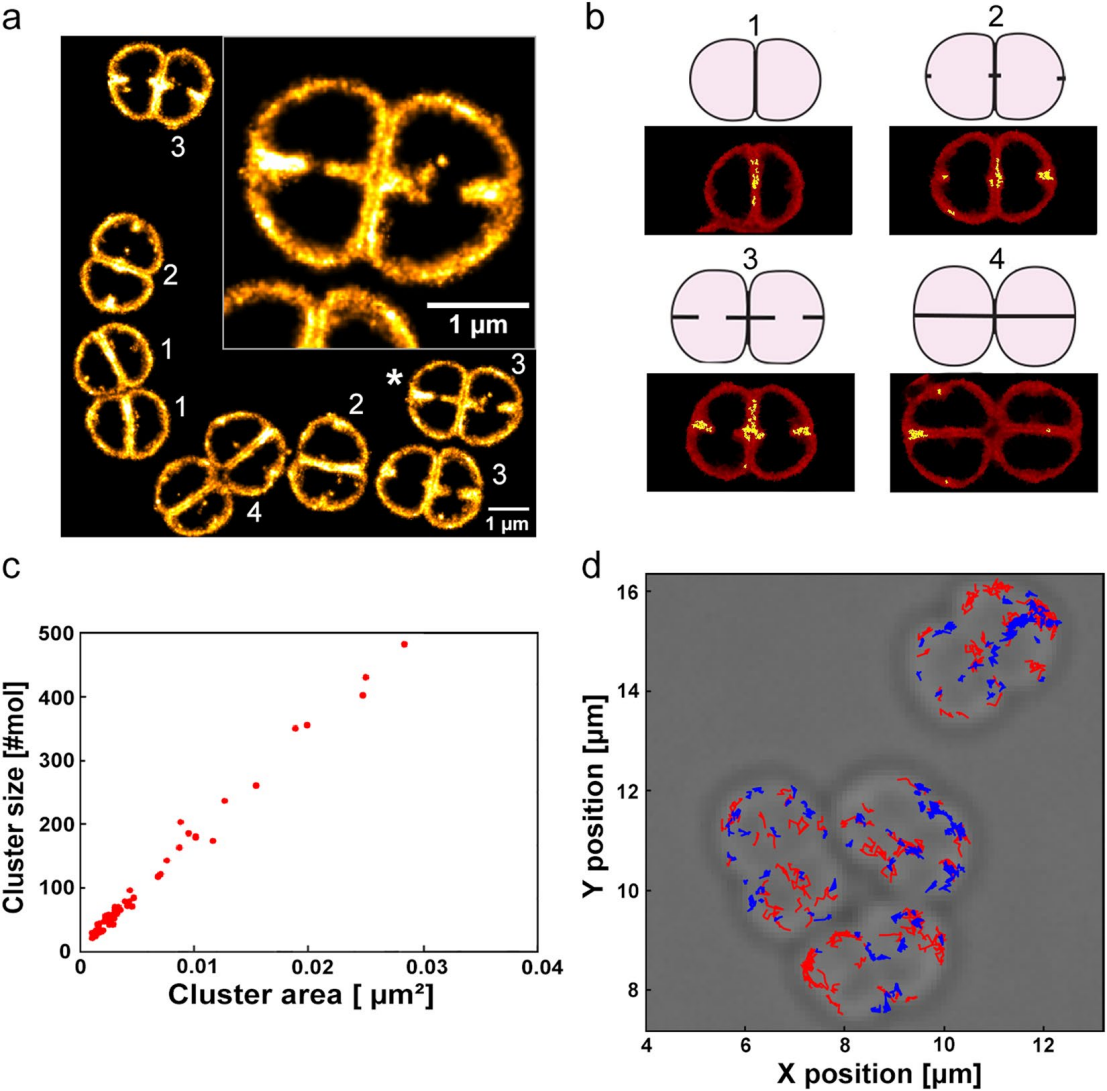
Cell division and septa formation in *D. radiodurans*. (**a**) Autoblinking-based PAINT image (15 ms frametime; 561 nm laser) of exponentially growing, live, unlabeled *D. radiodurans* cells. Bacteria are seen at different stages of their division cycle shown schematically in (**b**). Scale bar: 1 μm. Inset: close-up view of a *D. radiodurans* diad (indicated with a *) in which the double-layered cell wall separating the two bacteria can clearly be seen. (**b**) Schematic representations of *D. radiodurans* morphology along its cell cycle. Representative bacteria in each stage are shown in red as Voronoï diagrams following cluster analysis of the image shown in (**a**). Identified clusters are highlighted in yellow. (**c**) Number of autoblinking molecules per cluster as a function of cluster area. (**d**) Individual tracks of autoblinking molecules superimposed on the corresponding brightfield image. The faster and slower diffusing molecules are shown in red and blue respectively.

Interestingly, by deliberately choosing sample preparation conditions favoring high levels of autoblinking, we could acquire remarkably well-defined images of *D. radiodurans* cell walls in which the double-layered internal septa can clearly be visualized (Fig. 8a). We hypothesize that the improved resolution and quality of these images result from the high density of autoblinking molecules allowing to acquire an image stack rapidly, thereby minimizing image blurring due to residual motion of the live cells.

### Insight into septum formation and cell wall structure in *D. radiodurans*

*D. radiodurans* is a spherical bacterium that divides in alternating perpendicular planes^41^. Our autoblinking-based SMLM images of the cell wall of live *D. radiodurans* provide unprecedented views of the morphology of these bacteria along their cell cycle (Fig. 8a and Supplementary Fig. S1). Four different stages can be distinguished, that provide snapshots of growing internal septa. In stage 1, *D. radiodurans* cells are in the form of diads with a single internal septum separating the two cells; in stage 2, foci at the positions of future septa become visible, perpendicular to the previous cell division plane; in stage 3, newly forming septa are closing; and in stage 4, these new septa are closed and *D. radiodurans* cells form tetrads that will rapidly separate into two diads for a new cell cycle.

Figure 8a clearly shows that the density of autoblinking molecules is not homogeneous throughout the cell wall. To further explore these heterogeneities, we performed a cluster analysis using the SR-Tesseler software^42^. We computed potential clusters using a threshold δ > 2δ_N_, where δ_N_ is the average autoblinking molecular density in the analyzed fields of view. Identified ‘clusters’ located preferentially within the internal septa of the bacteria and at sites of new septa formation (Fig. 8b). Their broad size distribution and constant molecular density (Fig. 8c) suggest that autoblinking molecules do not form clusters with a preferential radius in *D. radiodurans*, but instead bind to septal regions at a faster apparent rate, likely as a result of a higher concentration of binding sites in the double-layered structure of the cell wall in these regions. In addition, as shown in Supplementary Fig. S2, the lower curvature of the cell wall at the septum also contributes to the increased apparent molecular density.

Interestingly, visual inspection of the fluorescent traces of the autoblinking molecules revealed that some of them appeared to be largely immobile, while others seemed to diffuse around the periphery of the cells. A few immobile molecules remained visible for more than 1 s (number of localizations >20; Supplementary Fig. S11), producing autoblinking “hotspots”. These observations hinted that autoblinking molecules may display several diffusion regimes depending on their localization within the cell wall, and potentially in relation with the different observed stages of cell division. To investigate this, several sptPAINT data sets were acquired and analyzed, in which the trajectories of individual autoblinking molecules were reconstituted (Fig. 8d). Cumulative probability distribution (CPD) analysis^43^ of more than 1000 individual tracks extracted from multiple datasets revealed that two distinct populations of molecules could be distinguished. No specific distribution of these two populations throughout the cell wall could be observed (Fig. 8d). Approx. half of the molecules displayed an average apparent diffusion coefficient of ~0.065 µm^2^/s and were clearly seen to move along the cell wall with a confinement radius of ~250 nm, whereas another half were nearly immobile, exhibiting an apparent diffusion coefficient of ~0.014 µm^2^/s that can be accounted for solely by their localization uncertainty (Supplementary Figs S12, S13 and Table S4).

Together, these data demonstrate that autoblinking is a powerful tool for imaging *D. radiodurans* cell walls and for following septa formation, but also for probing the structural and chemical complexity of the multiple layers composing these unusual cell walls.

## Discussion

In this work, we have confirmed that many bacteria show a tendency for autoblinking, which should be carefully taken into consideration when analyzing SMLM data. We have discovered that *D. radiodurans* and *D. deserti* stand out amongst the tested bacteria, by exhibiting particularly strong autoblinking. Our data demonstrate that the autoblinking phenomenon is caused by transient binding of fluorescent molecules originating mostly from the growth medium to bacterial cell walls in a PAINT-based regime. Agarose was also found to be a source of autoblinking molecules to a lesser extent, in contrast to the impurities commonly observed on uncleaned coverslides, which did not contribute to autoblinking levels. We hypothesize that autoblinking molecules might have a common origin linked to contact with plastic materials. This could explain why older stocks of chemicals result in increased levels of autoblinking. Until the exact nature of the autoblinking molecules is revealed, it cannot be excluded that they are composed from a heterogenous pool of molecules with different spectral and photophysical properties. Finally, we almost never detected such molecules as single emitters in the extracellular medium. This could be explained by their fast diffusion in the liquid phase and/or by their possible fluorogenicity whereby their fluorescence would be enhanced upon binding to specific environments such as lipid bilayers or agarose.

The strong binding in the case of *D. radiodurans* and *D. deserti* probably results from the unusual cell wall composition of these microorganisms. *D. radiodurans* stains Gram-positive, but its cell wall does not resemble that of classical Gram-positive bacteria, as it is composed of five different layers^21,29^ (Supplementary Fig. S3). Although our reconstructed images of *D. radiodurans* cell walls do not permit us to directly identify which layer of the cell wall is preferentially bound by autoblinking molecules, several observations indicate that it is likely to be the plasma membrane: (i) autoblinking molecules are found in both the external cell wall and the internal septa of *D. radiodurans* cells, suggesting that they are most likely not binding to the carbohydrate and pink envelope layers that are only found on the external periphery of the bacterial diads or tetrads, (ii) the density of autoblinking molecules is increased in the internal septa in agreement with the peptidoglycan and plasma membrane layers being doubled in these regions, and (iii) treatment of cells with the detergent, Triton X-100, but not with lysozyme, which degrades the peptidoglycan layer, disrupts the localization of the autoblinking molecules to the cell wall. Although *D. deserti’*s cell wall has not been as thoroughly characterized as *D. radiodurans’*, it has also been shown to possess a similarly dense cell wall, which may also favor the binding and trapping of autoblinking molecules within a lipid-containing layer.

Autoblinking in bacteria in general, and in *D. radiodurans* in particular, offers both advantages and disadvantages. The possibility to achieve label free nanoscale imaging of the bacterial cell wall is of definite potential interest. Precisely delineating the boundaries of bacterial cells being imaged using super-resolution techniques is critical for image analysis and data interpretation. Differential interference contrast or brightfield images superimposed onto fluorescence nanoscopy images typically provide inaccurate cell boundaries (see Fig. 2d), in particular due to high sensitivity of such images to the axial position of the sample relative to the focal plane. In addition, such images do not allow distinguishing changes in cell wall structure or newly forming septa during cell division. Observing such morphological features typically requires extra manipulation of the sample such as addition of a lipid targeting dye that is suitable for single-molecule localization imaging. Autoblinking, in contrast allows achieving label-free imaging of the cell wall of live *D. radiodurans* cells at sub-diffraction resolution. In this way, unprecedented images of *D. radiodurans* could be obtained, providing snapshots of internal septa formation at various stages of the cell cycle. Moreover, analysis of autoblinking data allowed us to observe clear heterogeneities in the distribution and dynamics of autoblinking molecules within the cell wall that reflect the complex nature of this essential cell barrier.

Autoblinking molecules may advantageously be used for sptPAINT, notably because they sparsely decorate the cell wall of bacteria in a seemingly endless manner. In *D. radiodurans*, the high-level of autoblinking in fact prevents the use of another exogenous dye to perform single-particle tracking experiments, because a too high density of labeling would be required to hide the contribution of autoblinking. In this bacterium, we observed two approximately equal populations of autoblinking molecules, one nearly immobile and one diffusing along the cell wall periphery with an apparent diffusion coefficient of 0.06 μm^2^/s. In *E. coli*, it was shown that different lipid binding dyes may exhibit different diffusion behaviors, and that the dye DiI-C12 displays two diffusion regimes, pointing at heterogeneities in the bacterial membrane^44^. Moreover, autoblinking molecules in *E. coli* were also recently found to diffuse with a diffusion coefficient close to 0.06 μm^2^/s^14^. Our results in *D. radiodurans* possibly suggest that heterogeneities in the plasma membrane of this bacterium also exist. However, we cannot exclude that this could also result from the possible inherent heterogeneity in the autoblinking molecules themselves. In addition, the absolute values of our diffusion coefficients should be taken with care, due to the relatively long frametimes that we used (30 ms)^45^, effects of diffusion in 3D^44^, and the difficulty to accurately extract several populations of molecules experiencing confined diffusion regimes by currently available software. Further work will be required to establish the full potential of the autoblinking phenomenon for sptPAINT.

In this study, we never totally silenced autoblinking, but we succeeded in minimizing it using well-defined sample preparation routines and carefully planned imaging schemes, taking into account the unusual photophysical characteristics of autoblinking molecules, in order to successfully image PTFP-labelled targets expressed in *D. radiodurans*. The thorough characterization of the autoblinking molecules, nonetheless, clearly reveals a single-molecule behavior that significantly overlaps with those of PTFPs typically used in PALM bacterial imaging such as PAmCherry. This makes the strict discrimination between them and PTFP-labeled targets challenging, particularly in the case of colocalization with cell wall associated low abundance target proteins. In such cases, it would be recommended to use alternative imaging protocols such as dSTORM, with bright organic fluorophores emitting light in the far-red region of the spectrum. However, in the future, resonant out-of-phase fluorescence microscopy^46^ or improvements in spectroscopic SMLM could enable differentiating autoblinking molecules and fluorescent proteins at the single molecule level. In general, the autoblinking phenomenon offers exciting prospects for multicolor semi-label free nanoscopy of bacteria.

## Methods

### Detailed methods are provided in Supplementary Methods

**Bacterial cultures:** *Deinococcus radiodurans* strains were grown at 30 °C in either TGY2X or in minimal medium as described previously^47^. *Deinococcus deserti* VCD115 strain was grown as described earlier^48^. *Bacillus subtilis* and *Escherichia coli* strains were grown at 37 °C in respectively TGY and LB medium. **Single-molecule imaging:** State-of-the-art cleaning protocols were used for sample preparation^3,49^. Images were acquired at 20 °C with a home-built PALM setup based on an Olympus IX81 microscope (Olympus) and equipped with diode-pumped solid-state lasers at 405 nm (CrystaLaser), 488 nm (Spectra-Physics), 561 nm (Cobolt) and 643 nm (Toptica Photonics). Fluorescence images were acquired with an Evolve 512 back-illuminated EMCCD camera (Photometrics) controlled by the Metamorph software (Molecular Devices). Sample drift was corrected in ImageJ using gold nanobeads (Sigma) deposited on the agarose pads. Autoblinking events were analyzed with the ThunderStorm analysis plugin^50^ in Fiji^51^. Single-molecule data were processed as described previously^35^. For spectral imaging, the optical set-up was built similarly to the set-up described by Zhang *et al*.^33^. Localization and evaluation of the spectral properties of the detected single molecules was done on a home-made software and compiled as a plugin of the MetaMorph software (Molecular Device).

## Electronic supplementary material

Supplementary Information

Supplementary Movie S1

## Data Availability

All data generated and analyzed in this study are either included in this published article (and its Supplementary Information files) or available from the corresponding authors on reasonable request.
