# Supplementary material for: Bacterial cell wall nanoimaging by autoblinking microscopy
Source: Sci Rep. 2018 Sep 19;8:14038. doi: 10.1038/s41598-018-32335-z (PMC6145920; doi:10.1038/s41598-018-32335-z)
Supplement: Supplementary file 1 — Supplementary Information [file 41598_2018_32335_MOESM1_ESM.pdf]

Bacterial cell wall nanoimaging by autoblinking microscopy

Kevin Floc'h[1], Françoise Lacroix[1], Liliana Barbieri[1], Pascale Servant[2], Remi Galland[3,4], Corey Butler[3,4], Jean-Baptiste Sibarita[3,4], Dominique Bourgeois[1*], Joanna Timmins[1*]

Supplementary Information

Supplementary Discussion

Supplementary Methods

Supplementary Tables S1-S4

Supplementary Movie S1

Supplementary Figures S1-S13

Supplementary References

Supplementary Discussion

Effect of the objective's depth of field on the observed width of the bacterial cell wall in 2-D super resolution images.

A careful observation of the *D. radiodurans* autoblinking images (Fig. 2c and e) suggests that the apparent resolution of these images is significantly affected by a projection effect of the curved 3-D cell wall onto the 2-D imaging plane. Such projection effect is not specific to *D. radiodurans* nor to the autoblinking process, but occurs for any 2-D super-resolution image of samples displaying a curvature along the optical axis that is significant within the objective depth of field. Thus, the effect is likely present in many super-resolved images of bacterial cell walls shown in the literature. Hence, we set out to perform simulations demonstrating the issue (Supplementary Fig. S2) and highlighting that our data are consistent with autoblinking molecules binding to very thin lipid-containing layers of the cell wall.

Using Matlab, we generated two different 3-D cylinders each decorated with 5000 randomly positioned fluorophores: one with a radius of curvature of 1.25 μm, close to the average radius of curvature of *D. radiodurans*, and one with a much larger radius of curvature of 5 μm. Both of these cylinders possessed a 10 nm thick wall. To perform the simulations, a homemade Matlab package was used[1] that allows generating localization microscopy data based on a comprehensive description of the microscope setup as well as the employed fluorophore's photophysics. The signal of each blinking fluorophore was thus

deformed according to the objective point spread function at its Z position, assuming a 1.49 NA objective exhibiting a depth of field of 500 nm. The outputs consisted of stacks of 10,000 images, similar to classical PALM stacks, which were subsequently processed in the same way as the other experimental data sets presented in this paper. The results presented in Supplementary Fig. S2 clearly show the expected effect, with the curved cylinder showing an effective thickness of ~80 nm and the straight cylinder showing a thickness of only ~40 nm. In Fig. S2e, the shape of the 1-D profile is clearly asymmetric on each side as a result of Z-projection. A similar shape can be observed in Fig. 2e, demonstrating the impact of the described effect.

Supplementary Methods

***Deinococcus radiodurans* strains**

A codon-optimized gene encoding PAmCherry was gene-synthesized (MWG Eurofins) and cloned into the plasmid p11559 for expression in *D. radiodurans* under the control of an IPTG-inducible p*Spac* promoter. Wild-type *D. radiodurans* cells (ATCC13939) were transformed with p11559-PAmCherry as described previously[2] and transformants were selected on TGY (Tryptone, Glucose, Yeast extract) agar plates containing 75µg.ml^{-1} spectinomycin. The Δ*crtI* strain (GY15501) and the genetically engineered strain of *D. radiodurans* expressing HU fused to PAmCherry (GY17031) were obtained by the tripartite ligation method[3]. Briefly, the gene encoding PAmCherry and the regions flanking the insertion site (3' end of *hu* gene, DR_A0065) were PCR amplified, and the three fragments were ligated together. *D. radiodurans* cells were then transformed by the ligation product and plated on selective medium containing 6 µg.ml^{-1} kanamycin, leading to allelic replacement on one genome copy. Because *D. radiodurans* is multigenomic, the transformant colonies were further streaked three times successively on selective medium to ensure that all copies of the genome had incorporated the foreign DNA.

Bacterial cultures

All *Deinococcus radiodurans* strains were grown at 30°C with shaking in either TGY2X or in minimal medium (MM) as described previously[4]. PAmCherry expression from the p11559 plasmid was induced by addition of 1mM IPTG to the cultures. For growth in MM, cells were initially grown in TGY2X and were transferred to MM at an optical density at 650nm (OD_{650}) of 0.2-0.4 and were incubated at 30ºC for a further 24h to reach OD_{650} ~0.6-0.8. This procedure was repeated several times to allow the cells to adapt to growth in MM. *Deinococcus deserti* VCD115 strain was grown in diluted TSB (tryptic soy broth) medium supplemented with trace elements at 30°C with shaking as described previously[5]**.** *Bacillus*

subtilis and *Escherichia coli* strains (BL21 and lycopene producing strain, BW-Lyco) were grown at 37°C with shaking in respectively TGY and LB medium.

Sample preparation for microscopy

Glass coverslides used for *D. radiodurans* imaging were treated in an ozone oven for at least 10 minutes. 1.75% low-melting agarose (Biorad) pads dissolved in MM were poured on a cover slide inside a frame made from double-faced tape. A glass coverslip was placed on top of the agarose pad in order to flatten the surface of the agarose while hardening. This coverslip was removed once the agarose had solidified. For imaging, bacterial cultures in exponential (OD_{650} ~0.3) or stationary phase (OD_{650} >2) were centrifuged 5 min at 3000 x g and were resuspended in 10µl of medium or washed in high purity PBS (GIBCO). For live cell imaging, 1µl of this cell suspension was deposited on the pads. The drop was spread over the surface of the pad by rotating the coverslip. A second coverslip was placed over the agarose pad containing the sample, thereby immobilizing the bacteria on the pad. For fixed-cell imaging, cells were fixed for 10 min at room temperature in the culture medium containing 3.7% formaldehyde and resuspended in high purity PBS (GIBCO) prior to deposition on the agarose pads or directly on coverslips. Minimal autoblinking levels were obtained when the cells were rinsed several times in high purity PBS (GIBCO). When desired, higher autoblinking levels could be obtained by depositing live cells in rich TGY medium directly on the agarose pads. To assess the levels of autoblinking in the growth media, 1µl of medium (TGY and MM) was deposited on the agarose pads along with gold nanobeads that were used to define the focus. The levels of autoblinking of our different samples were determined by evaluating the number of blinking events per μm^2 per 1000 frames. For samples with bacteria, only the surface covered by cells was taken into account for these calculations, while for the growth media control samples, the full imaged area was used. Cell wall staining with Nile Red (Sigma) was achieved by incubating the bacteria with 15 nM Nile Red for 5 min prior to depositing the cells on agarose pads. Digestion of the carbohydrate and peptidoglycan layers of *D. radiodurans* was achieved by incubating exponentially growing bacteria in 4 mg/ml lysozyme for 30 min at 37ºC. Disruption of the lipid containing layers of the cell wall was achieved by incubating cells in 0.1% Triton X-100 at 20ºC for 4 min. The cells were then rinsed and deposited on agarose pads for imaging.

Image acquisition and analysis

Wide-field illumination was achieved by focusing the laser beams to the back focal plane of a 100× 1.49-numerical-aperture (NA) oil immersion apochromatic objective lens (Olympus). The intensity and time sequence of laser illumination at the sample were tuned by an acousto-optical tunable filter (AOTF;

Quanta Tech). Laser beam profiles were recorded before each experiment using a coverslip uniformly marked with a fluorescent dye so as to retrieve the power densities along the laser profile. Near circular polarization of the laser beams was ensured by inserting a polychromatic quarter-wave plate downstream the AOTF. All PALM/PAINT data were collected with continuous 561-nm light illumination using a frametime of 50 ms, except for two-color acquisitions where the PAmCherry channel was acquired with frametimes of ~5 ms. High-resolution images of *D. radiodurans* cell walls (Fig. 8a) were acquired on cells exhibiting particularly high levels of autoblinking. In this case, the frametime used was 15 ms, allowing to collect a stack of images in a short time, thereby minimizing image blurring due to moving or growth of the cells. 5 to 20 cells were imaged per field of view. Typically, for each sample 2 or 3 fields of view on the same agarose pad were imaged and at least three independent experiments were performed on different days. Sample drift was corrected in ImageJ using gold nanobeads (Sigma) deposited on the agarose pads nearby the bacteria.

SptPAINT data were acquired with continuous 561 nm light illumination, at low power (130 W/cm²) using a frametime of 30 ms. Importantly sptPAINT experiments could not be performed with Nile Red on *D. radiodurans* as the relatively high concentration needed to 'mask' autoblinking was incompatible with the sparsity of events required by this method to follow single-molecule tracks unambiguously. The Trackmate plugin for ImageJ was used to localize the particles in each frame and to connect the coordinates into trajectories[6]. Simple LAP (Linear Assignment Problem) Tracker generated the tracks, with a maximal allowed linking distance set to 200 nm and a maximal frame interval between two spots to be bridged set to 2. Only tracks that contained more than four points were exported into MATLAB for further processing. The TrackArt MATLAB software[7] was used to analyze trajectories and determine cumulative probability distributions and apparent diffusion coefficients. Trajectories shorter than 10 frames or with a minimal individual mean square displacement (MSD) fit "R^2" values below 0.9 were filtered out. TrackArt estimated the relative error on the diffusion coefficients by calculating the diffusion coefficients of 10 randomly chosen sets of trajectories containing 50% of all trajectories. The standard deviation for these 10 diffusion coefficients was calculated and the relative error was estimated by extrapolating the standard deviation to the full dataset. Imposing only one population of diffusing molecules resulted in a deviation of the fit of the CPDs exceeding the uncertainty of the data.

Cluster analysis was performed with SR-Tesseler[8] using the coordinates of autoblinking molecules as input data (*i.e.*, after merging of localizations). Potential clusters were computed using a threshold $\delta > 2\delta_N$, where δ_N was the average localization density inside the bacterial contour.

Photophysical analysis

Localizations were clustered using defined space and time limits enabling, for each cluster, to calculate a fluorescence time trace. The time traces were analyzed based on the iterative method of Li *et al*[9], which allows extracting the single molecules possibly belonging to a single cluster. Analysis of on-time, off-time and apparent bleach-time histograms was then performed to extract the corresponding rates. The absence of significant change in these rates when the applied laser power was increased led us to conclude that the overall apparent blinking behavior of autoblinking molecules was essentially driven by light-independent transient binding to the D. *radiodurans* cell wall. Thus final fluorescence time traces were obtained by applying an arbitrary small cut-off value of 0.1 s to rescue artifactual short blinks due to missed detections. Values reported in Supplementary Table S1, S2 and S3 were extracted from these traces.

Spectral single-molecule imaging and analysis

Two infinity-corrected microscope objectives, 1 (Nikon, 100x OI – NA 1.49) and 2 (Nikon, 60x WI – NA 1.27), were placed on each side of the sample and aligned to focus on the same plane. Illumination lasers (405 nm; 488 nm; 561 nm; 646 nm) were combined into a single mode optical fiber after passing through an acousto-optic tunable filter (AOTF) and sent into an azimuthal TIRF illumination system (Roper, iLas[2]). The laser beam was then sent into the sample via objective 1 (Nikon, 100x – NA 1.49) and a Quad Band filter cube (AHF, F66-04TN). Fluorescence signal was collected on one side by objective 1 and directed to a EMCCD camera (Photometrics, Evolve™ 512) through the Quad Band filter on path 1. On the other side, objective 2 (Nikon, 60x – NA 1.27) collected the fluorescence signal, which was filtered by a long pass filter (AHF, F76-567 – 568 long pass filter) and a Quad Band Notch filter (AHF, F57-406) and directed to a second EMCCD camera through path 2. Path 2 was composed of a 4f system providing 1.5x magnification in which a 10° wedge prism (Thorlabs, PS814-A) was inserted at the Fourier plane to transform the spectral property of the detected fluorescence into a spatial shift onto the EMCCD camera. Calibration of the spectral dispersion and resolution of path 2 was achieved by imaging Tetraspeck beads (0.1 µm in diameter, Invitrogen) coated with 4 different dyes with well-separated spectra (excitation and emission peaks: 360/430 nm; 505/515 nm; 560/580 nm; 660/680 nm). Tetraspeck beads were absorbed on a glass coverslip at low density and mounted in PBS between two coverslips using a custom sample holder and excited successively with a 488 nm laser, a 561 nm, and a 642 nm laser. For each excitation wavelength, the beads were localized on both cameras and the

displacement induced by the prism in path 2 was measured. This enabled us to determine that the spectral shift induced by the 10° wedge prism inserted in path 2 was of 11.2 nm/pixels.

For analysis, a first calibration step was performed to superimpose the fields acquired in the two channels. Mono-color beads (Nano-diamond – Adámas Nanotechnologies, NDNV100nmMd10ml) were displaced along an 8x8 grid covering the whole acquisition field of view and an image was acquired on both channels for each grid position. The bead positions were then determined by Gaussian fitting separately on each channel and the field transformation from path 2 to path 1 was determined by fitting a second order polynomial function. The error of superposition of the two channels was less than 30 nm after field transformation. The spectral properties of the detected autoblinking single molecules were then determined: the localization of each single molecule was done on path 1 by 2D Gaussian fitting and then reported to the Channel 2. This defines for each detected single molecule a region of interest on channel 2 where the single molecule spectrum has been acquired. Averaging those spectra for hundreds of localizations enables to eventually reconstruct the spectrum of the detected autoblinking molecules.

It must be noted that, because each single-molecule spectrum is convolved with the point spread function (PSF) of the spectral detection path, reconstructed spectra are broadened by ~30 nm. Other factors such as low photon counts, pixilation noise, spatially varying local background and precision of the calibration also affect the spectral resolution, especially when a low number of single molecules are detected, as for example is the case for the agarose pad spectrum in Fig. 4.

Supplementary Tables

Table S1: Photophysical properties of autoblinking molecules and PAmCherry fluorescent proteins in fixed *D. radiodurans* under constant 0.8kW/cm² 561nm laser .

	Autoblinking	PAmCherry
Mean number of emitted photons per localization	335±63	352±19
Photon Counts per molecule*	563±31	1150±70
Dissociation Rate [s^{-1}]	17.1±0.9	
Bleaching Rate [s^{-1}]		8.8±0.9
Blinking ON-OFF Rate [s^{-1}]		6.8±0.7
Blinking OFF-ON Rate 1 [s^{-1}]		16.4±0.8
Blinking OFF-ON Rate 2 [s^{-1}]		2.0±.0.3
Fraction of molecules with Rate 1		0.4±0.0

* In the case of autoblinking, the "photon counts" relates to the integrated number of recorded photons during the binding time and in the case of PAmCherry relates to that number until photobleaching.

Table S2. Photophysical properties of autoblinking molecules in response to illumination with 488nm, 561nm and 647nm lasers at a constant laser power of 0.8kW/cm².

	Autoblinking		
	488nm	561nm	647nm
Relative density of localizations	0.71	1	0.09
Mean Photon Counts per molecule	381±46	563±31	329±85
Dissociation Rate [s^{-1}]	23.8±2.8	17.1±0.9	18.9±2.9

Table S3. Photophysical properties of autoblinking and Nile Red molecules under increasing 561nm laser illumination power.

	0.2 kW/cm²		0.8 kW/cm²		1.6 kW/cm²	
	Autoblinking	Nile Red	Autoblinking	Nile Red	Autoblinking	Nile Red
Mean Photon Counts per molecule	330	290	560	470	600	610
Apparent Dissociation Rate [s^{-1}]	19	21	17	22	18	19

Table S4: Diffusion coefficients and confinement radii of the two populations of autoblinking molecules.

	Population 1	Population 2
D_{app} diffusion coefficient (µm²/s)	$(6.35\pm0.39)\times10^{-2}$	$(1.42\pm0.09)\times10^{-2}$
Confinement radius (nm)	249	86
Fraction of the whole populations	0.48±0.01	0.52±0.01

Supplementary Movie

Movie S1: Extract (500 out of 50,000 images) of the stack used for autoblinking-based imaging of the *D. radiodurans* cell presented in Fig. 2. Images were acquired with 50 ms frametimes under continuous 0.8kW/cm² 561nm laser.

Supplementary Figures

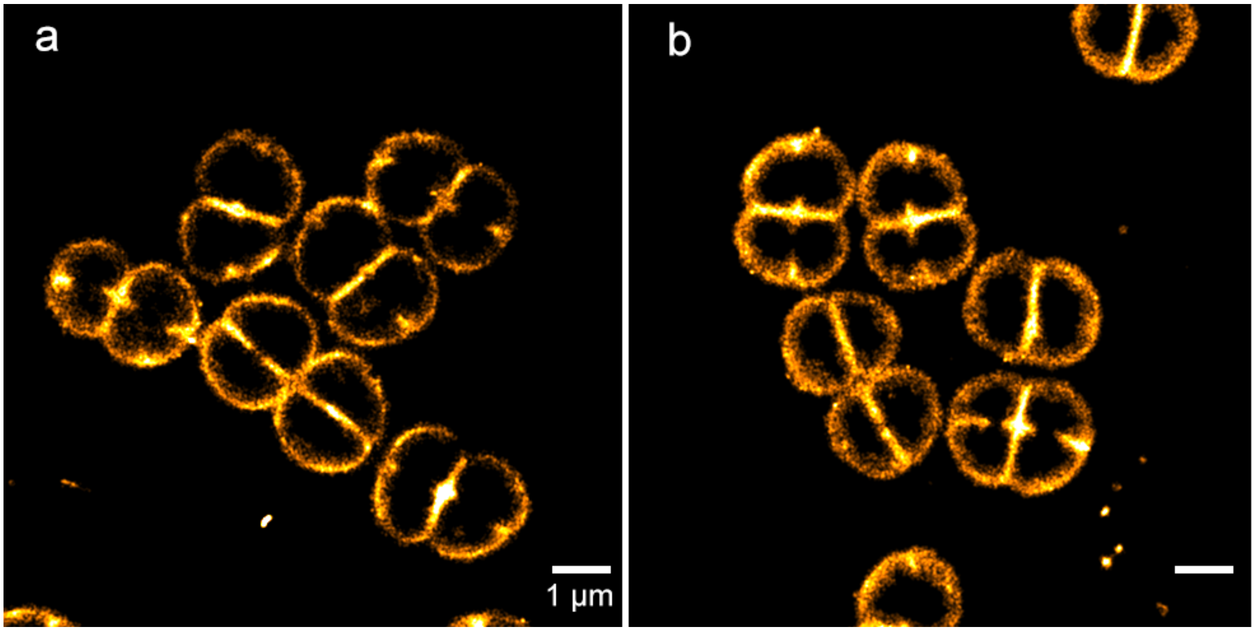

Figure S1. Examples of super-resolved images of live *D. radiodurans* acquired using either (a) autoblinking or (b) Nile Red labeling. Scale bar: 1µm. Images were acquired with a 50 ms frametime under continuous 0.8kW/cm² 561nm laser. The autoblinking reconstruction (a) was prepared using 30,000 frames, whereas the Nile Red reconstruction (b) was prepared using 15,000 frames.

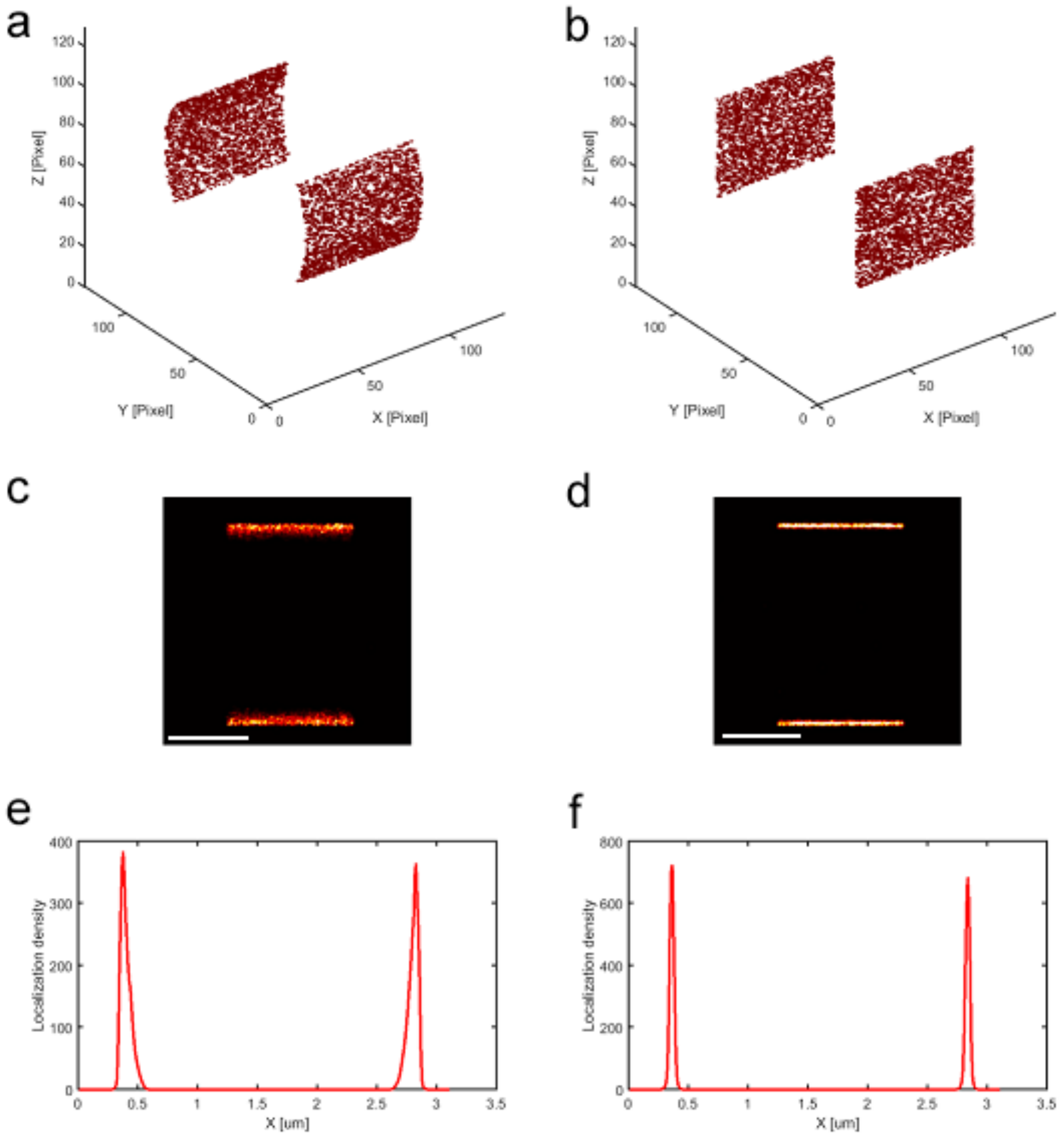

Figure S2. Effect of cell wall curvature on apparent image resolution. (a,b) 3-D views of 5,000 fluorophores decorating Z-sections (1.25 mm overall height) of 10-nm thick cylinders of high (1.25 μm) and low (5 μm) radius of curvature. (c-d) PALM images reconstituted from data sets simulated with Matlab, using the samples shown in (a-b), and an objective depth of field of 500 nm. Photophysical parameters of typical blinking fluorophores were used in the simulations, achieving a mean localization precision of 20 nm. Scale bar: 1 μm. (e-f) 1-D projection profiles from (c-d) along the vertical dimension, highlighting the loss of resolution resulting from sample curvature along the optical axis.

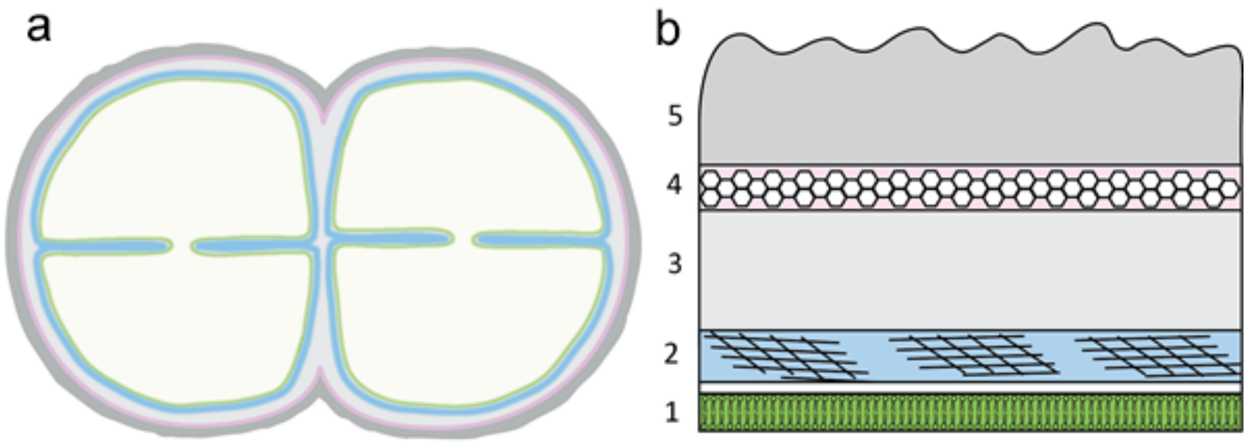

Figure S3. Schematic representation of the complex cell wall structure of *D. radiodurans* (a), composed of five distinct layers (b): 1, plasma membrane (green); 2, peptidoglycan layer (blue); 3, interstitial layer (light grey); 4, S-layer or backing (pink) composed of proteins, lipids and carotenoids; 5, carbohydrate layer (dark grey). The thickness of *D. radiodurans* cell wall is estimated to be between 75 and 150nm[10,11].

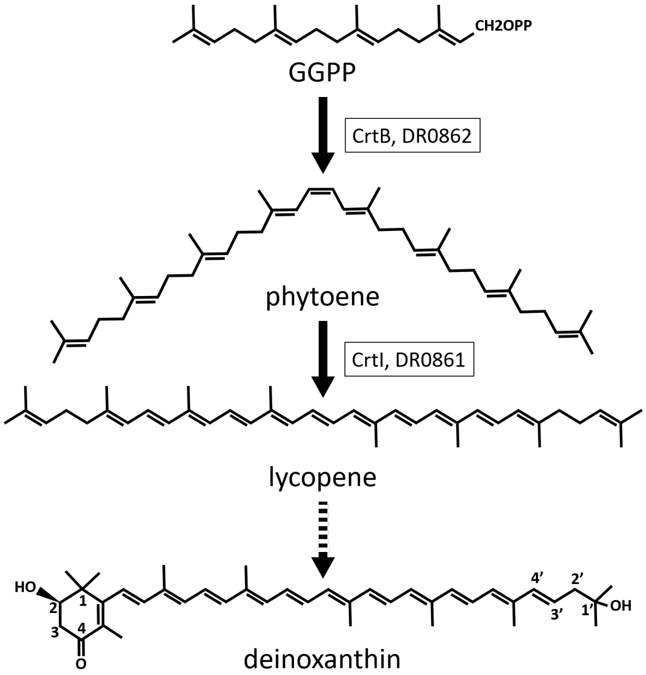

Figure S4. Schematic representation of the deinoxanthin carotenoid biosynthetic pathway of *D. radiodurans*. Bold arrows indicate a single step; discontinuous arrows refer to multiple steps. Abbreviations: GGPP, geranylgeranyl-pyrophosphate. A Δ*crtB* strain of *D. radiodurans* accumulates GGPP precursor, while a Δ*crtI* mutant accumulates phytoene by-product.

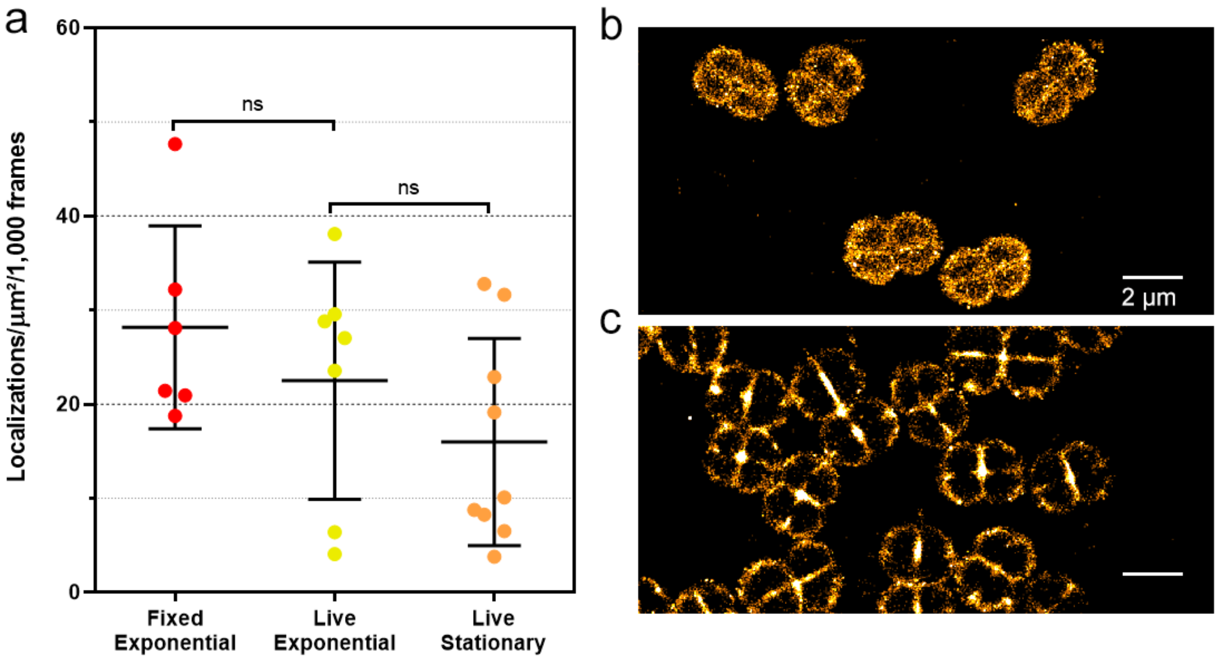

Figure S5. (a) Autoblinking levels (localizations/µm²/1000 frames) in fixed or live wild-type *D. radiodurans* cells, in exponential (OD=0.3) or stationary (OD>4) growth phases and grown in rich TGY medium. Images were acquired with a 50 ms frametime under continuous 0.8kW/cm² 561nm laser. Individual data points correspond to the autoblinking levels derived from a given stack of images. Means and standard deviations are plotted in the graph. (b)-(c) Examples of super-resolved images of fixed *D. radiodurans* (b) and live, stationary phase cells (c). Cell fixation leads to a reduction in the size of the bacteria and deteriorates the quality of the cell wall labeling. In both cases, reconstructions were prepared using 15,000 frames acquired with a 50 ms frametime and under continuous 0.8kW/cm² 561nm. Scale bar: 2µm.

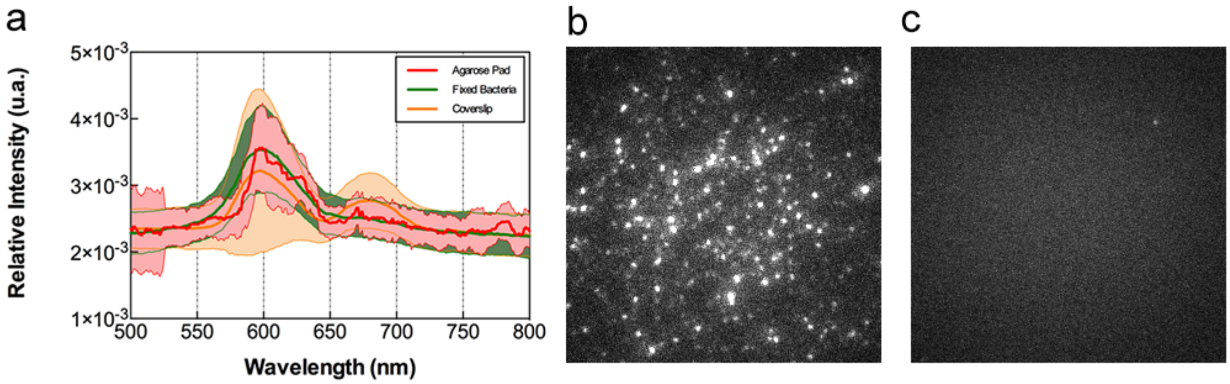

Figure S6. Effect of ozone-treatment of the glass coverslides. (a) Spectral imaging of single molecules found at the surface of the glass coverslides (no ozone treatment; orange), of the agarose pad (red) or at the periphery of fixed *D. radiodurans* cells (green). An additional peak centered around 675 nm is seen for blinking molecules at the surface of glass coverslides. (b) Numerous blinking molecules are seen at the surface of untreated coverslides. (c) Incubation of the glass coverslides for 10 min in an ozone oven is sufficient to remove most of these blinking molecules.

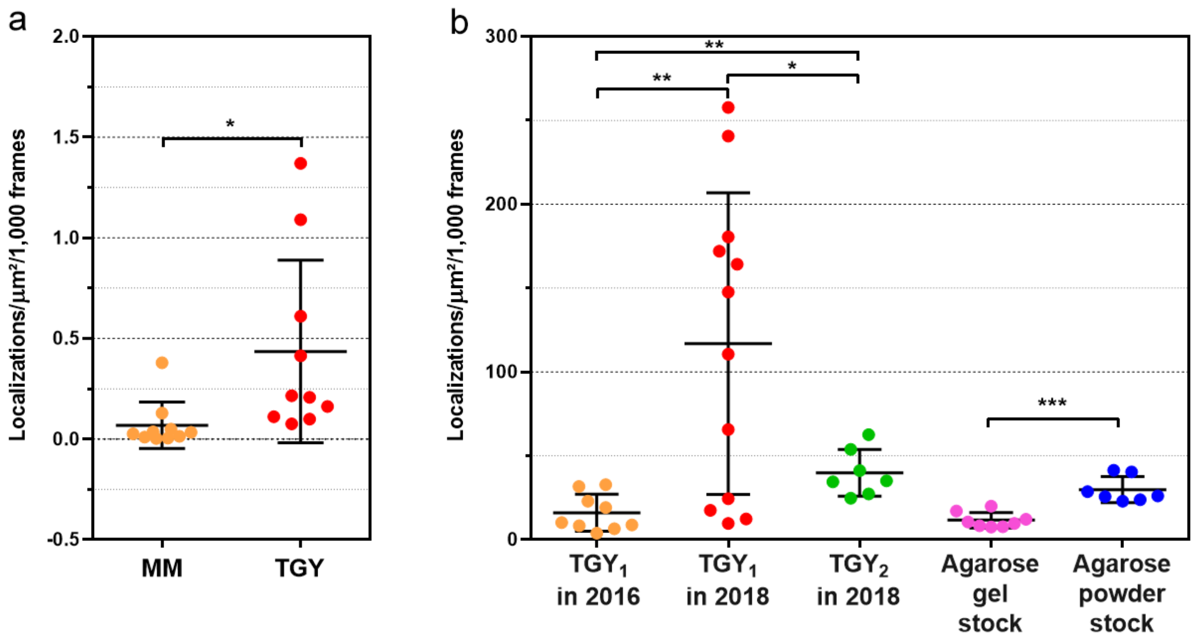

Figure S7. Autoblinking levels in growth medium and effect of conditioning and storage time of chemicals on autoblinking levels in *D. radiodurans*. (a) Autoblinking levels (localizations/µm²/1000 frames) in minimal medium (MM) and in TGY medium deposited directly on agarose pads in the absence of bacterial cells. The number of localizations are extracted from the entire field of view and not exclusively from the areas covered by cells as is the case in Figures 3, 5, S5a and S7b. (b) Autoblinking levels (localizations/µm²/1000 frames) in live *D. radiodurans* cells grown in different sources of TGY medium. TGY_1 in 2016: experiments performed in 2016 on cells grown in TGY medium purchased in 2016 (stock 1). TGY_1 in 2018: experiments performed in 2018 on cells grown in TGY medium purchased in 2016 (stock 1). TGY_2 in 2018: experiments performed in 2018 on cells grown in TGY medium purchased in 2018 (stock 2). Autoblinking levels (localizations/µm²/1000 frames) in live *D. radiodurans* cells deposited on agarose pads prepared either from agarose stored as a gel in a glass container (Agarose gel) or from agarose powder stored in a plastic container (Agarose powder). (a)-(b) Images were acquired with a 50 ms frametime under continuous 0.8kW/cm² 561nm laser. Individual data points correspond to the autoblinking levels derived from a given stack of images. Means and standard deviations are plotted in the graph.

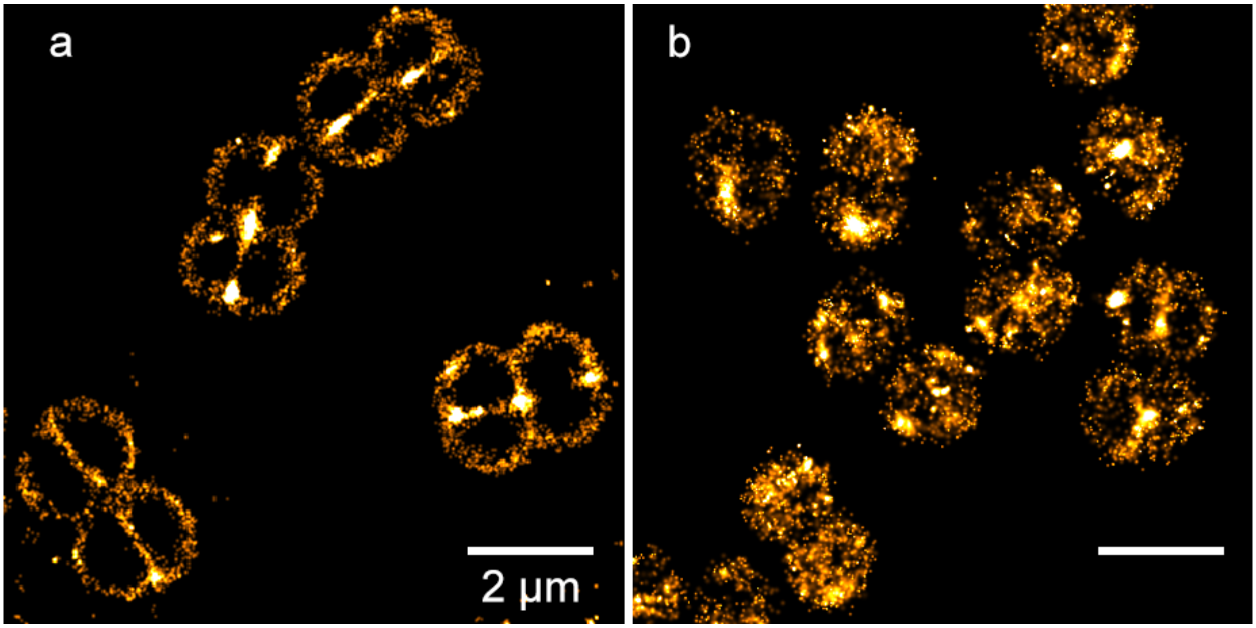

Figure S8. Effect of lysozyme and Triton X-100 treatments on autoblinking distribution in live, unlabelled *D. radiodurans*. (a) Images of cells treated for 30 minutes at 37°C with 4mg/ml lysozyme, (b) images of cells treated with 0.1% Triton X-100 for 4 minutes at 20ºC. In both cases, reconstructions were prepared using 5,000 frames acquired with a 50 ms frametime and under continuous 0.8kW/cm² 561nm. Scale bar: 2 µm.

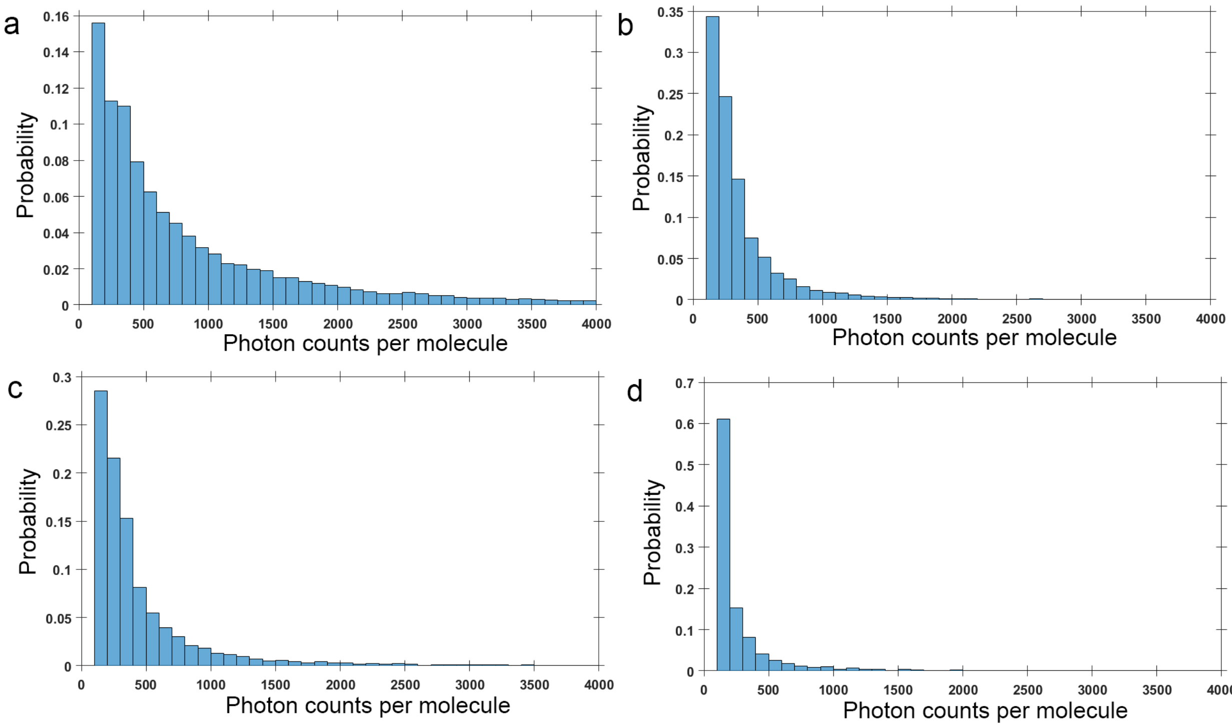

Figure S9. Histogram distributions of the photon counts per molecules for PAmCherry molecules (a) under 561 nm illumination and for autoblinking molecules (b)-(d) under 488 nm (b), 561 nm (c) or 643 nm (d) illumination. All data were acquired with a constant laser power of 0.8kW/cm².

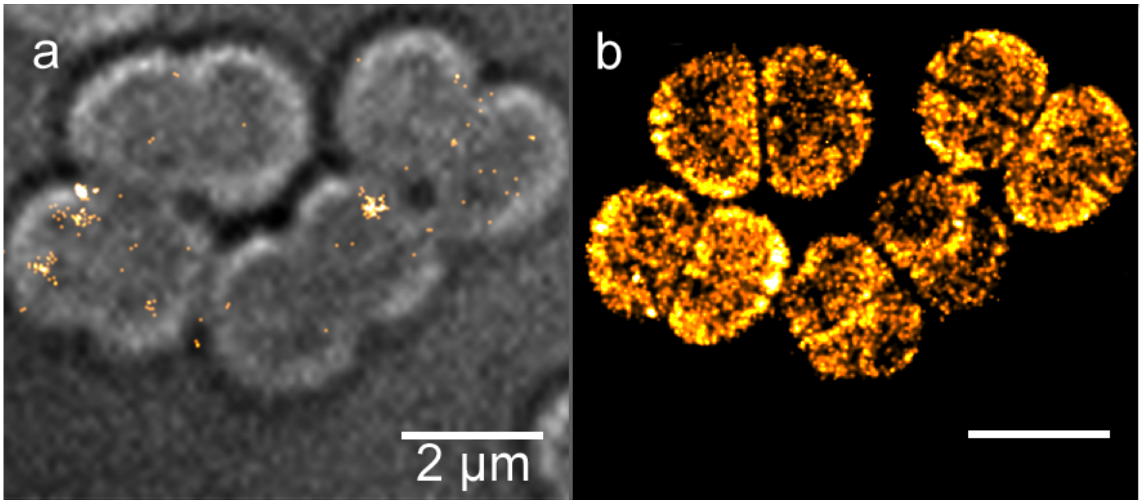

Figure S10. PALM imaging of photo-activated fluorescent proteins in autoblinking bacteria. (a)-(b) 4000 image stacks of autoblinking (a) and PAmCherry (b) in *D. radiodurans* expressing free PAmCherry, acquired with ~5 ms frametimes, under continuous 0.8kW/cm² 561nm laser, and either without (a) or with (b) 0.4 W/cm² 405nm laser. Scale bar: 2 µm.

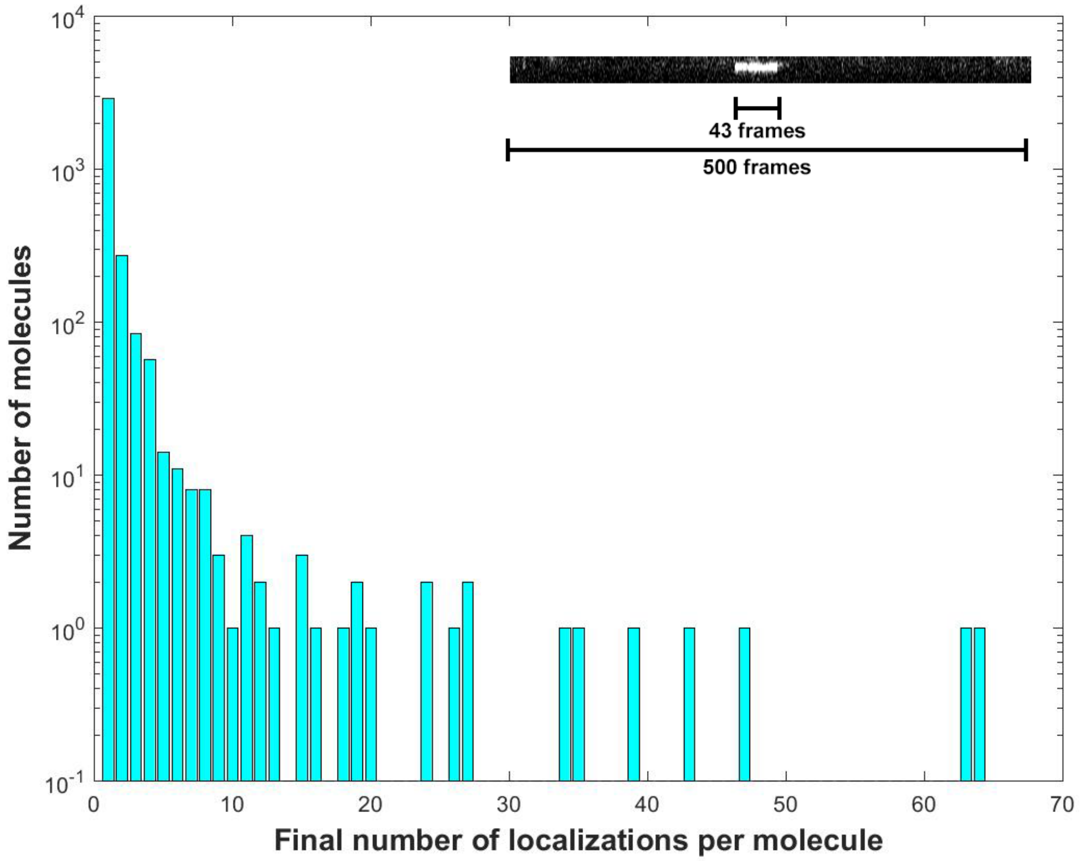

Figure S11: Number of localizations per autoblinking molecules during a 10,000 frame acquisition. Inset: example of a kymograph showing the localization of a given molecule in 43 consecutive frames.

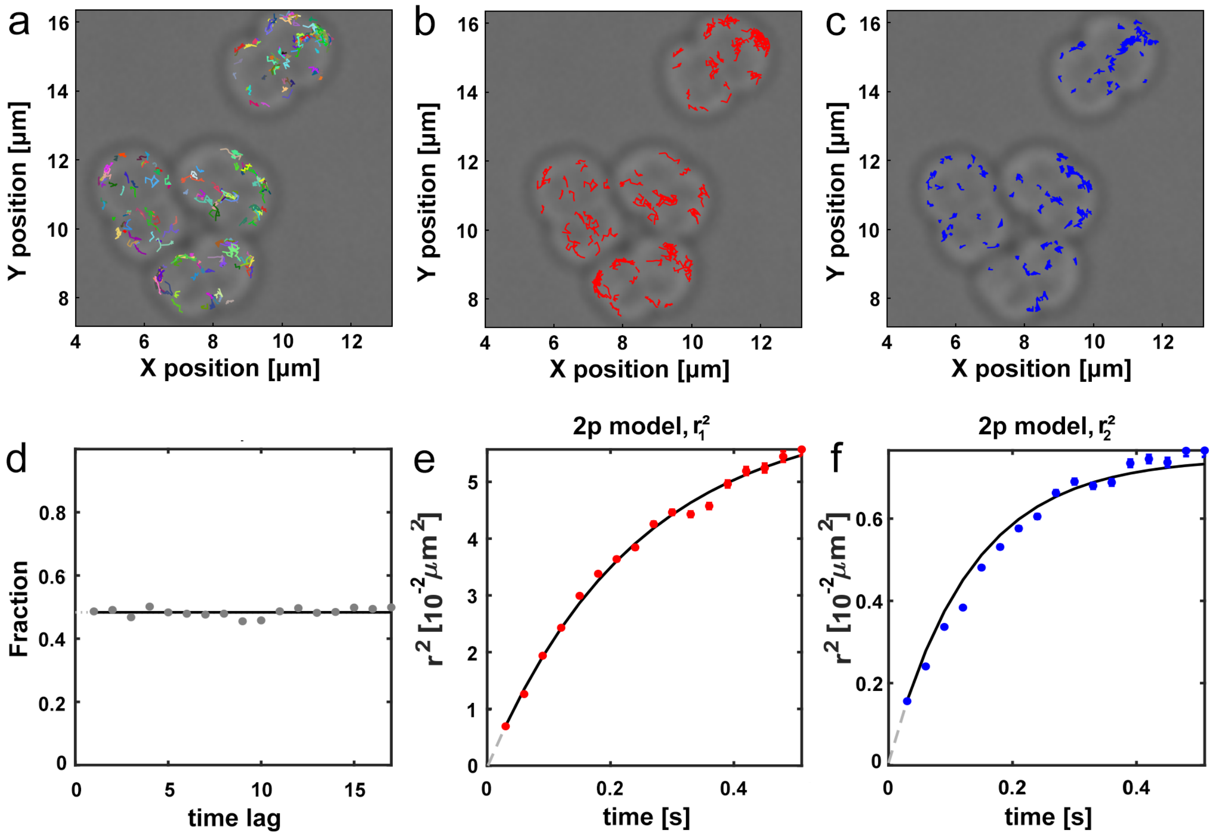

Figure S12. Single-particle tracking analysis of autoblinking molecules. (a)-(c) Individual trajectories of autoblinking molecules superimposed on the corresponding brightfield image. Faster and slower diffusing molecules are colored respectively red (b) and blue (c). (d) Fraction of the slower population over the faster diffusing population. (e)-(f) Mean square deviation (MSD) plots of the faster population (e) and the slower population (f). (Δt = 30 ms). Both populations are confined.

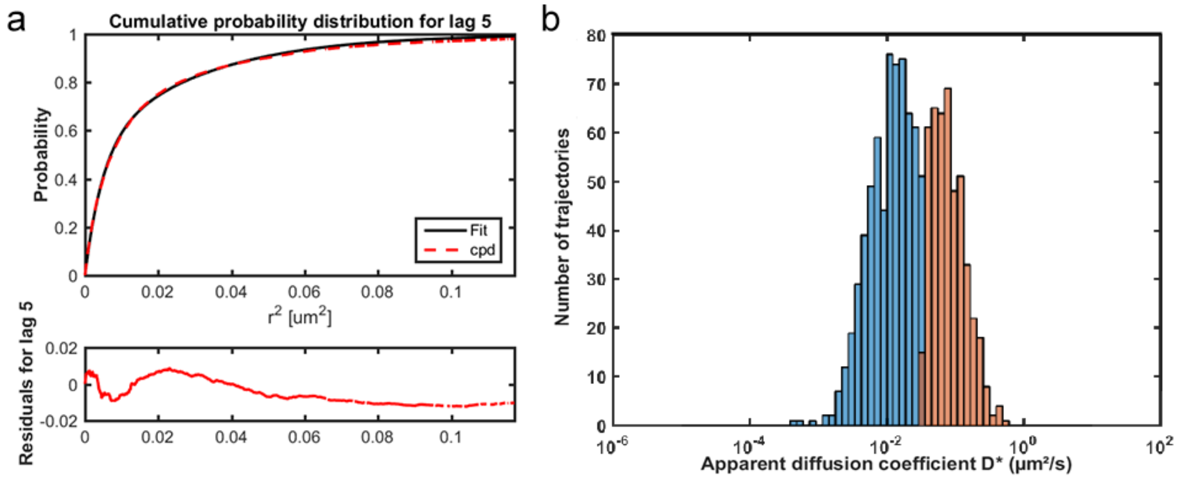

Figure S13. (a) Cumulative probability distribution (CPD) of mean square displacements and residuals for the fit of the two-population model of autoblinking molecules. Distributions and fits are presented only for the fifth time-lag (Δt = 30 ms). The two-species model described in this manuscript is retrieved from the fit of the CPD. (b) Distribution of apparent diffusion coefficients. As an alternative analysis of the single particle tracks, the distribution of the apparent coefficients was fitted with a simple Gaussian mixture model. The results are consistent with the two-population model obtained from CPD analysis, with a slower population (blue, 61% of the whole population, with D=0.015 µm²/s) and a faster one (orange, 39% of population with D=0.065 µm²/s).

Supplementary References

1. Avilov, S. *et al.* In cellulo evaluation of phototransformation quantum yields in fluorescent proteins used as markers for single-molecule localization microscopy. *PloS one* **9**, e98362 (2014).
2. De Almeida, C. B., Coste, G., Sommer, S. & Bailone, A. Quantification of RecA protein in Deinococcus radiodurans reveals involvement of RecA, but not LexA, in its regulation. *Molecular Genetics and Genomics* **268**, 28–41 (2002).
3. Mennecier, S., Coste, G., Servant, P., Bailone, A. & Sommer, S. Mismatch repair ensures fidelity of replication and recombination in the radioresistant organism Deinococcus radiodurans. *Molecular genetics and genomics* **272**, 460–469 (2004).
4. Venkateswaran, A. *et al.* Physiologic Determinants of Radiation Resistance inDeinococcus radiodurans. *Applied and environmental microbiology* **66**, 2620–2626 (2000).
5. De Groot, A. *et al.* Deinococcus deserti sp. nov., a gamma-radiation-tolerant bacterium isolated from the Sahara Desert. *International journal of systematic and evolutionary microbiology* **55**, 2441–2446 (2005).
6. Tinevez, J.-Y. *et al.* TrackMate: An open and extensible platform for single-particle tracking. *Methods* **115**, 80–90 (2017).
7. Matysik, A. & Kraut, R. S. TrackArt: the user friendly interface for single molecule tracking data analysis and simulation applied to complex diffusion in mica supported lipid bilayers. *BMC research notes* **7**, 274 (2014).
8. Levet, F. *et al.* SR-Tesseler: a method to segment and quantify localization-based super-resolution microscopy data. *Nature methods* **12**, 1065–1071 (2015).
9. Lee, S.-H., Shin, J. Y., Lee, A. & Bustamante, C. Counting single photoactivatable fluorescent molecules by photoactivated localization microscopy (PALM). *Proceedings of the National Academy of Sciences* **109**, 17436–17441 (2012).
10. Rothfuss, H., Lara, J. C., Schmid, A. K. & Lidstrom, M. E. Involvement of the S-layer proteins Hpi and SlpA in the maintenance of cell envelope integrity in Deinococcus radiodurans R1. *Microbiology* **152**, 2779–2787 (2006).
11. Slade, D. & Radman, M. Oxidative stress resistance in Deinococcus radiodurans. *Microbiology and molecular biology reviews* **75**, 133–191 (2011).
