# Supplementary figures and images for: Bacterial cell wall nanoimaging by autoblinking microscopy
Source: Sci Rep. 2018 Sep 19;8:14038. doi: 10.1038/s41598-018-32335-z (PMC6145920; doi:10.1038/s41598-018-32335-z)

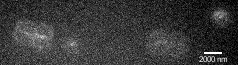

Supplement: Supplementary file 2 — Supplementary Movie S1 [file 41598_2018_32335_MOESM2_ESM.gif]
